# A Systematic Review of Therapeutic Approaches Used in Experimental Models of Interstitial Cystitis/Bladder Pain Syndrome

**DOI:** 10.3390/biomedicines9080865

**Published:** 2021-07-22

**Authors:** Tadeja Kuret, Dominika Peskar, Andreja Erman, Peter Veranič

**Affiliations:** Institute of Cell Biology, Faculty of Medicine, University of Ljubljana, 1000 Ljubljana, Slovenia; tadeja.kuret@mf.uni-lj.si (T.K.); dominika.peskar@mf.uni-lj.si (D.P.); andreja.erman@mf.uni-lj.si (A.E.)

**Keywords:** interstitial cystitis, bladder pain syndrome, therapeutic approaches, experimental models, in vitro, ex vivo, in vivo

## Abstract

Interstitial cystitis/bladder pain syndrome (IC/BPS) is a multifactorial, chronic bladder disorder with limited therapeutic options currently available. The present review provides an extensive overview of therapeutic approaches used in in vitro, ex vivo, and in vivo experimental models of IC/BPS. Publications were identified by electronic search of three online databases. Data were extracted for study design, type of treatment, main findings, and outcome, as well as for methodological quality and the reporting of measures to avoid bias. A total of 100 full-text articles were included. The majority of identified articles evaluated therapeutic agents currently recommended to treat IC/BPS by the American Urological Association guidelines (21%) and therapeutic agents currently approved to treat other diseases (11%). More recently published articles assessed therapeutic approaches using stem cells (11%) and plant-derived agents (10%), while novel potential drug targets identified were proteinase-activated (6%) and purinergic (4%) receptors, transient receptor potential channels (3%), microRNAs (2%), and activation of the cannabinoid system (7%). Our results show that the reported methodological quality of animal studies could be substantially improved, and measures to avoid bias should be more consistently reported in order to increase the value of preclinical research in IC/BPS for potential translation to a clinical setting.

## 1. Introduction

Interstitial cystitis/bladder pain syndrome (IC/BPS) is a multifactorial, chronic bladder disorder of unknown etiology, generally characterized by discomfort or pain in the bladder and the surrounding pelvic region, associated with increased urinary frequency, urgency, and nocturia [1]. IC/BPS is more frequent in women compared to men with an estimated prevalence of 45–300 per 100,000 women and 8–30 per 100,000 men [2,3,4]. However, the occurrence of IC/BPS is likely to be underreported due to the complexity of the disease, a variety of different and nonspecific clinical symptoms and signs, and a lack of standardized diagnostic criteria [5,6]. To date, there is no effective therapeutic option available for patients with IC/BPS, and the disease represents an enormous financial burden for the individuals and the economy as a whole [7].

In general, IC/BPS can be categorized into two major subtypes, mainly based on the bladder histological findings [8]. The first type or “classical” IC/BPS with Hunner’s lesions (i.e., mucosal lesions accompanied by abnormal capillary structures) is characterized by more severe bladder-centric symptoms, reduced bladder capacity, histological signs of epithelial denudation, inflammatory infiltrates, and edema, while IC/BPS without Hunner’s lesions has no obvious bladder etiology, features minimal histological changes, and is frequently accompanied by common systemic comorbidities (“bladder-beyond” pain) [9,10].

Regardless of the IC/BPS subtype, the overall etiology and pathophysiology remain elusive with many different hypotheses proposed over the years, including injury of the bladder epithelium and increased barrier permeability, neurogenic inflammation with mast cell infiltration, and possible autoimmune involvement [11,12]. One of the most common characteristics found in bladder biopsies from IC/PBS patients is denudation or thinning of the bladder urothelium, a specialized type of epithelial tissue that lines the wall in the majority of the urinary tract and plays an important role as a permeability barrier against toxic substances from the urine [13]. In IC/BPS patients, the barrier function is compromised due to various reasons, including the reduction of the glycocalyx layer, consisting of glycoproteins and proteoglycans [14], the disassembly of tight junctions with deregulated expression of certain tight junction proteins (zonula occludens-1 (ZO-1), occludin, and claudins 1, 4, and 8), and reduced expression of specific transmembrane proteins uroplakins [15,16,17]. The compromised urothelial barrier results in the leakage of urine solutes, such as potassium and urea into the lamina propria, leading to the activation of inflammatory response with increased urothelial release of signaling molecules (e.g., acetylcholine (ACh), adenosine triphosphate (ATP), nitric oxide (NO)) and proinflammatory mediators, such as interleukins (IL)1, IL6, and IL8, tumor necrosis factor alpha (TNFα), and nerve growth factor (NGF), as well as increased nerve fiber density and inflammatory (mast cell) infiltrates, which ultimately contribute to urgency and pain [15,18,19]. Proinflammatory mediators sensitize afferent nerve terminals by activating transient receptor potential (TRP) channels resulting in the release of neuropeptides (e.g., calcitonin gene-related peptide (CGRP) and substance P) that induce mast cell degranulation and further stimulate the release of proinflammatory mediators, leading to a perpetual cycle of inflammation and pain [20,21,22,23].

Although various therapeutic options exist for patients with IC/BPS, all of them aim to relieve the symptoms and there is no treatment nor combination of treatments currently available that would be consistently successful in alleviating clinical symptoms and ensuring long-term efficacy. The American Urological Association (AUA) guidelines [24] recommend a stepwise therapeutic approach, in which the first-line therapy includes patient education with daily behavior modification and lifestyle changes. Physical therapy, oral administration of pentosan polysulfate (PPS) or antihistamines, and intravesical application of heparin, lidocaine, or dimethyl sulfoxide (DMSO) constitute second-line therapy. Third-line therapy requires cystoscopy and hydrodistension, while neuromodulation and intravesical injection of botulinum toxin A (BTX-A) are considered as a fourth-line therapy. If a patient does not respond to any of the therapeutic agents, surgical intervention (cystectomy) is needed [24].

The currently available and recommended therapy options for IC/BPS patients are based mostly on empirical studies and suffer from low efficacy. Hence, research on IC/BPS is focusing on the development and evaluation of novel therapeutic options. Since the pathophysiology of IC/BPS is not yet well understood, the development of definitive therapeutic modalities is significantly compromised, and, despite promising preclinical results of various therapeutic agents, only a low percentage reached clinical trials. This might be related to the lack of suitable and validated experimental models that would be able to replicate all aspects of IC/BPS complexity, as well as the inadequate methodological quality of experimental in vivo models and incomplete reporting of relevant information according to published guidelines of animal research [25].

**Figure 1:**
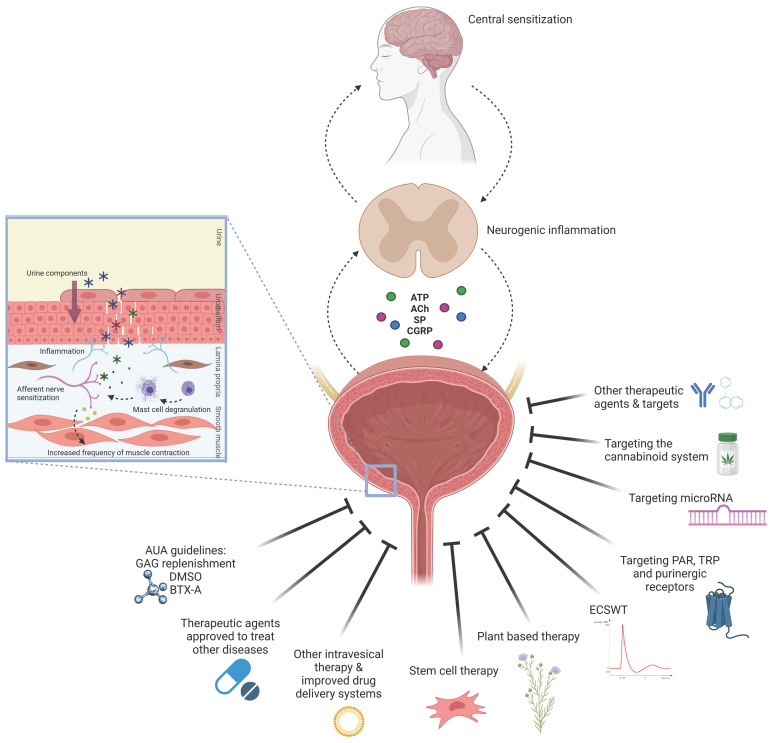
Schematic illustration of IC/BPS pathology and therapeutic approaches evaluated in experimental in vitro, ex vivo, and in vivo models of IC/BPS. Legend: ACh, acetylcholine; ATP, adenosine triphosphate; AUA, the American Urological Association; BTX-A, botulinum toxin A; CGRP, calcitonin gene-related peptide; DMSO, dimethyl sulfoxide; ECSWT, extracorporeal shock wave therapy; GAG, glycosaminoglycan; IC/BPS, interstitial cystitis/bladder pain syndrome; PAR, protease-activated receptors; SP, substance P; TRP, transient receptor potential channels; solid lines indicate the therapeutic approaches for treatment of IC/BPS; dashed arrows indicate proposed sequence of events in IC/BPS pathophysiology. The Figure 1 was created using Biorender.com.

The review aims to give insight into the commonly used experimental in vitro, ex vivo, and in vivo models for IC/BPS, as well as to summarize and discuss the therapeutic approaches used in these models, explain their mechanism of action, and estimate their translational potential. We also aimed to report on the methodological quality of included studies and evaluate whether sufficient measures to avoid the risk of bias were undertaken. The therapeutic approaches identified were categorized into five groups: (i) therapeutic agents currently recommended by AUA guidelines to treat IC/BPS, (ii) therapeutic agents currently approved to treat other diseases, (iii) other intravesical therapy and improved drug delivery systems, (iv) novel emerging therapeutic options and targets, which include stem cell and extracorporeal shock wave therapy (ECSWT), plant-derived agents, or novel potential targets, such as protease-activated receptors (PAR), purinergic receptors, TRP channels, microRNAs, and activation of the cannabinoid system, and (v) other therapeutic agents and targets (Figure 1).

## 2. Methods

### 2.1. Search Strategy

A comprehensive literature review was conducted using PubMed, Scopus, and Web of Science databases to identify articles exploring therapeutic options in in vitro, ex vivo, and in vivo experimental models of IC/BPS. We used the following search terms: ((“interstitial cystitis” OR “bladder pain syndrome” OR “IC/BPS”) AND (“in vitro” OR “ex vivo” OR “in vivo” OR “animal” OR “models”) AND (“therapy” OR “treatment”)) in different combinations. Only full-text articles in English published from 1 January 2000 until 31 May 2021 were included. As the relationship between IC/PBS and other dysfunctional bladder syndromes in human patients (including the overactive bladder) is less well confirmed, this review is limited to those studies based only on experimental models of IC/BPS.

### 2.2. Inclusion and Exclusion Criteria and Data Extraction

Articles were reviewed in a two-stage process. The first stage included screening the titles and abstracts of all identified articles. Reviews, editorials, case reports, conference proceedings, notes, and articles not written in English were excluded. Additional exclusion criteria were irrelevant articles describing other diseases and not IC/BPS, articles not including therapeutic agents, and articles not describing an experimental model of IC/BPS. During the second stage, full texts of the remaining studies were evaluated. The reference list of the most relevant studies was also screened to identify any other potentially eligible studies. Two reviewers (T.K. and D.P.) independently assessed the full-text papers to determine if they met the inclusion criteria and selected the final articles to be included in this study. For in vitro and ex vivo studies, we extracted information regarding the experimental design of the study (type of cells used, type, concentration, and time of stimulation and therapy, major findings, and outcome). For in vivo studies, information was extracted for aspects of methodological quality (see below) and experimental design (animal number, species and strain, type, concentration, time and route of administration of IC induction and treatment agent, main findings, and outcome).

### 2.3. Methodological Quality and Risk of Bias

To determine the methodological quality of published in vivo studies, we defined a 12-point checklist based on published ARRIVE guidelines describing the minimum information that all scientific publications reporting research using animals should include [26]. We specifically focused on the study design (number of animals and experimental groups), experimental animals (species and strain, sex, age, and weight), detailed description of housing and husbandry, and detailed description of the experimental procedure, as well as reporting on measures to avoid the risk of bias (e.g., randomization, sample size calculations, blinding of investigator/caretaker, and blinding of outcome assessment).

## 3. Results and Discussion

**Figure 2:**
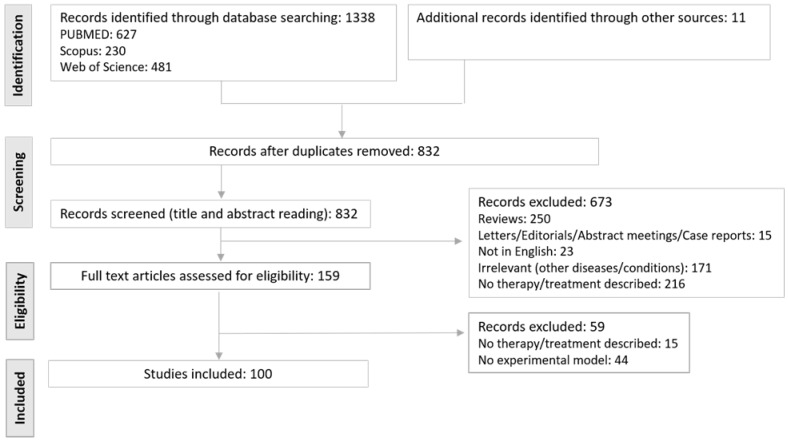
Flow diagram of study search and selection.

The electronic database (PubMed = 627; Scopus = 230; Web of Science = 481) and reference list search (*n* = 11) resulted in 1349 articles, of which 159 remained after the removal of duplicates and title/abstract screening. Finally, after assessing the full-text articles for eligibility, a total of 100 full-text articles were included in the present review. A flow diagram of the search and selection process is shown in Figure 2. Seven of the 100 included studies (7%) reported on in vitro models, five (5%) studies used ex vivo models, and 77 (77%) studies included in vivo models. Eleven (11%) studies included in vivo models in combination with in vitro (*n* = 10) or ex vivo (*n* = 1) models.

### 3.1. Experimental Models of IC/BPS

#### 3.1.1. In Vitro and Ex Vivo Models

Since the most consistently described findings in the bladders of IC/PBS patients include abnormalities in the urothelium [27], the majority of identified in vitro models (15/18; 83%) studied either primary urothelial cells, isolated/explanted from human or animal bladders or different urothelial cell lines (i.e., HTB2, HTB4). Most commonly, protamine sulfate (PS), TNFα, lipopolysaccharide (LPS), or H_2_O_2_ was used in in vitro models to induce urothelial dysfunction and mimic the proinflammatory environment observed in the bladders of IC/BPS patients. Ex vivo models included whole-bladder preparations (5/6; 83%) or bladder detrusor muscle strips (1/6; 17%), isolated from experimental animals. Whole bladders or muscle strips, mounted in organ baths, were stimulated chemically with carbachol, ACh, ATP, capsaicin ( TRPV1 receptor agonist) or KCl, or electrically, similar to triggering bladder contractions in vivo. These models were used to evaluate changes in bladder contraction activity induced by pathologic conditions (e.g., acute injury with HCl, H_2_O_2_, or acrolein), and to explore the nature of neurotransmission and sensitization of afferent pathways [28]. A summary table with the characteristics of each article describing in vitro and ex vivo models is provided (Table S1, Supplementary Materials).

#### 3.1.2. In Vivo (Animal) Models

In the present review, all of the in vivo studies (*n* = 88) were conducted on either mice or rats. According to Birder and Andersson, animal models of IC can be categorized into three subtypes, i.e., bladder-centric models, models with complex mechanisms, and stress-induced/natural models [29]. Most of the identified in vivo studies used bladder-centric models (76/88; 86%) with cyclophosphamide (CYP) being the predominant toxic substance for IC induction (29/88; 33%), followed by HCl (9/88; 10%), PS (6/88; 7%), LPS (5/88; 6%), or a combination of different toxins (11/88; 12%). Only a small number of reviewed studies incorporated more complex IC models, such as autoimmune models using immunization of wild-type or transgenic animals for IC induction (7/88; 8%), and stress-induced IC models (5/88; 6%). The majority of in vivo experiments included acute IC (55/88; 63%), while models of chronic IC, characterized by the treatment with bladder-toxic substances for more than 3 days or with more complex mechanisms of induction were described in 34% (30/88) of the reviewed studies (Table S2, Supplementary Materials). Three studies (3%) included both acute and chronic IC models. The most commonly evaluated outcomes of IC induction were nociceptive behavior and mechanical allodynia of the animals (e.g., with the application of von Frey monofilaments), urodynamic parameters with cystometry or void spot assay, and the extent of inflammation in bladder tissues (e.g., histology, immunohistochemistry, qPCR) or urine (levels of secreted proinflammatory mediators). Most of the studies exploited female rodents (77/88; 87%), while male animals were included in 9% of the studies (8/88).

### 3.2. Types of Treatment Evaluated in Experimental Models

**Figure 3:**
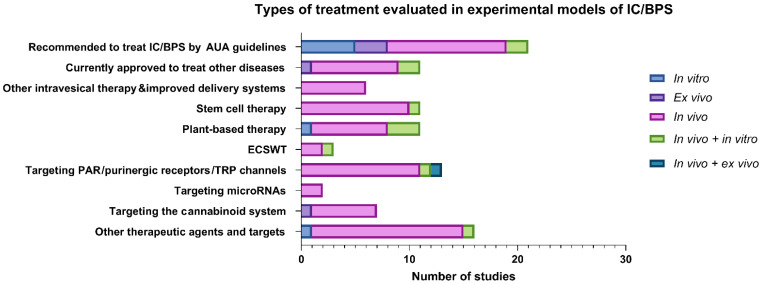
A summary of included experimental models evaluating different types of treatment for IC/BPS. Legend: AUA, the American Urological Association; ECSWT, extracorporeal shock wave therapy; IC/BPS, interstitial cystitis/bladder pain syndrome; PAR, protease-activated receptors; TRP, transient receptor potential.

The majority of identified articles included in the present review reported on experimental models, evaluating therapeutic agents currently recommended to treat IC/BPS by AUA guidelines (21/100; 21%), followed by therapeutic agents currently approved to treat other, most commonly chronic inflammatory diseases (11/100; 11%) and improved systems for intravesical drug delivery (6/100; 6%). More recently published articles evaluated therapeutic approaches using stem cells (11/100; 11%), plant-derived agents (10/100; 10%), and ECSWT (3/100, 3%). Novel potential drug targets for IC/BPS identified were PAR (6/100; 6%), purinergic receptors (4/100; 4%), TRP channels (3/100; 3%), microRNAs (2/100; 2%), and activation of the cannabinoid system (7/100; 7%), while other agents and targets (15/100; 15%) included hydroxyfasudil, vitamin D3, growth factors, and adhesion molecules (Figure 3).

#### 3.2.1. Therapeutic Agents Recommended by AUA Guidelines for Treatment of IC/BPS

##### Glycosaminoglycan Replenishment Therapy

According to the hypothesis that damage to the glycosaminoglycan (GAG) layer is among the main causes of IC/BPS symptoms, infusions of exogenous GAG biopolymers (e.g., hyaluronic acid (HA), chondroitin sulfate (CS), and heparin), and PPS intravesically into the bladder have been used in clinical practice for over two decades [30]. Several in vitro mechanistic studies that evaluated GAG replenishment treatment have been published recently, showing the ability of GAGs to decrease urothelial permeability and restore the barrier function; however, confounding results exist regarding their anti-inflammatory effects (Table S1, Supplementary Materials). To evaluate the effect of CS on the barrier function after induction of urothelial damage, Rozzenberg et al. used terminally differentiated porcine urothelial cells, which are morphologically and functionally comparable with the same types of cells in a normal human urothelium. Treatment with CS significantly accelerated the recovery of the barrier function 7 h after acute damage with PS [31]. Rooney et al. showed that high-molecular-weight HA significantly decreased TNFα- and PS-induced IL8 and IL6 production, increased sulfated GAG production, and decreased trans-epithelial permeability without altering tight junction protein expression in the HTB4 urothelial cell line [32]. This was later confirmed by Stellavato et al., showing that HA and CS, alone or in combination, were able to decrease IL6 and IL8 expression, as well as re-establish the expression of ZO-1 in TNFα-treated urothelial cell lines [33]. In contrast, the follow-up study in 2020 revealed that none of the commercially available GAG formulations containing HA or HA with CS were able to attenuate the TNFα-induced production of IL8 and IL6, the expression of GAG synthesis enzymes, or markers of tissue remodeling and pain [34]. Later on, Rooney et al. also reported on a newly developed biphasic system combining cross-linked and native HA in a 1:1 ratio that was able to reduce permeability, while at the same time did not alter the production of proinflammatory cytokines in HTB2 cells [35]. The significant recovery in various cystometric parameters following HA treatment was shown in vivo in H_2_O_2_-induced IC in female Wistar rats. HA recovered inter-contraction interval, maximal voiding pressure, and the number of pelvic afferent and efferent nerve activities to near-normal levels by directly scavenging H_2_O_2_ or OH^−^ activity and decreasing bladder ATP and ACh levels [36]. The immediate effect of intravesical CS on the restoration of bladder permeability and reduced recruitment of inflammatory cells to the suburothelial space was shown in HCl-induced IC in BALB/c mice and Sprague-Dawley (SD) rats [37,38]. Additionally, male SD rats, given a premix of PPS and low-molecular-weight toxic factor, derived from the urine of healthy individuals, showed significantly lower numbers of non-voiding contractions compared to the untreated group [39]. Since the linear GAGs, commonly used in IC/BP therapy, are not able to mimic the normal urothelial hydrophilic surface consisting of a thick glycocalyx layer with large numbers of bound water molecules [40], novel GAG-replenishment strategies are being developed. Greenwood-Van Meerveld et al. reported on restored bladder function and reduced bladder permeability by intravesical instillation of recombinant human proteoglycan 4 (lubricin, rhPRG4), a highly hydrophilic glycoprotein with anti-inflammatory properties in PS-induced IC in female SD rats [41]. The same research group also tested a novel high-molecular-weight GAG biopolymer (“SuperGAG”) that was more effective in restoring bladder function and relieving pain compared to CS [42]. Another emerging class of therapeutic GAGs involves semi-synthetic GAG-ethers (SAGE) offering both mucosal restoration and potent analgesic and anti-inflammatory effects. For example, SAGE GM-0111 was tested by several groups demonstrating attenuation of inflammation [43,44,45]. These novel GAGs offer improved protection of the damaged urothelium, but still encounter many limitations, such as poor urothelial binding and consequent fast clearance with micturition. The synthetic polymer drug delivery systems offer a better accumulation of GAGs, but can potentially weaken normal bladder function by reducing bladder capacity or causing bladder outflow obstruction (BOO) [43].

##### Dimethyl Sulfoxide (DMSO)

In addition to PPS, a 50% *w*/*w* aqueous solution of DMSO (both recommended as a second-line therapy) is the only drug approved by the FDA for treating IC/BPS [24]. The mechanism of action of DMSO in IC/BPS is not entirely known; however, it is thought to be a combination of anti-inflammatory effects, nerve blockade, and smooth muscle relaxation [46]. Melchior et al. reported that DMSO at concentrations greater than 35% completely inhibits ex vivo bladder contractions, stimulated by the electrical field, ACh, or membrane depolarization [47]. The anti-inflammatory effect of 50% DMSO was shown in URO-OVA mice with activated OT-1 splenocyte-induced acute autoimmune inflammation and URO-OVA/OT-1 transgenic mice with spontaneously developed chronic IC. Three consecutive intravesical DMSO treatments reversed edema and hyperemia, as well as decreased the number of infiltrating CD8^+^ T cells. A significant downregulation in mRNA levels of proinflammatory mediators (MCP1, IL6, IFNγ, NGF, and TNFα) in acute IC was also observed [48]. Moreover, intravesical instillation of 50% DMSO in adult female Wistar rats with PS-induced acute IC significantly reduced edema, vascular congestion, and polymorphonuclear (PMN) count that persisted for 7 days after treatment. However, mild inflammation with PMN infiltrate and transient edema was provoked in DMSO-instilled normal bladders [49]. These findings might aid in the explanation of the occurrence of urethral irritation/pain, which is the most frequently reported side-effect (48% of patients) of DMSO instillation [50].

##### Botulinum Toxin A

Botulinum toxin A (BTX-A) is a potent neurotoxin produced by the bacterium *Clostridium botulinum* [51], currently approved by the FDA for the treatment of neurogenic detrusor muscle overactivity and refractory overactive bladder [52,53]. Due to the ability of BTX-A to inhibit ACh release from nerve fibers, resulting in muscle contractions, as well as prevent sensory nerves sensitization and inflammation, its use has been extended in urology also to treat IC/BPS, and it is currently recommended as a fourth-line therapy by AUA guidelines [24,54]. BTX-A application significantly decreased ATP- and capsaicin-induced neuronal activity in an ex vivo model of isolated rat bladders, as determined by decreased release of the sensory neuropeptide calcitonin gene-related peptide (CGRP) [55]. The ability of BTX-A to inhibit the neuropeptide release (CGRPH and substance P) was subsequently confirmed in bladders from normal adult male rats with acute or chronic IC [56]. BTX-A pretreatment of male rats with CYP-induced IC also reduced ATP release from the urothelial side of bladder preparations, as well as suppressed bladder hyperactivity, non-voiding contraction frequency, and COX-2 and EP4 expression [57,58]. Concurrently, these shreds of evidence suggest that the effects of BTX-A on bladder sensory actions might result from a combined inhibition of sensory neurotransmitter release and through modulation of purinergic pathways [57].

#### 3.2.2. Therapeutic Agents Currently Approved to Treat Other Diseases

Several therapeutics approved to treat different chronic pain, inflammatory, and allergic diseases have been evaluated in experimental models of IC/BPS. For example, antihistamines cetirizine and ranitidine significantly reduced chronic pelvic pain allodynia in experimental models of autoimmune IC in BALB/cJ mice [59]. Recently, Grundy et al. discovered that histamine induces mechanical hypersensitivity ex vivo by interacting with histamine H1 receptor and TRPV1, which was blocked in the presence of pyrilamine [60]. Montelukast, a leukotriene D4 receptor antagonist, used to prevent and treat asthma, re-established uroplakin distribution and tight junction protein expression and decreased inflammatory cell infiltration in PS-induced IC in Wistar albino rats [61]. In a mouse model of IC induced by CYP, administration of carbenoxolone, clinically prescribed to treat digestive ulcers and inflammation, prevented bladder inflammatory changes and urothelial injury, decreased micturition frequency, and increased micturition volume. Further in vitro analysis showed that carbenoxolone reduced CYP metabolite acrolein-induced injury of urothelial cells, isolated from normal mice bladders by decreasing the expression of TRPV4 channels and reducing TRPV4-mediated oxidative stress [62]. Another drug used to treat gastritis, rebamipide, decreased inflammatory cell infiltration, reduced levels of TNFα, IL1β, and IL6, recovered protein expression of uroplakin 3A, accelerated the repair of the damaged urothelium, and suppressed bladder overactivity and nociception in HCl-induced IC in SD rats [63]. Anti-inflammatory hydroxychloroquine (a TLR7/9 antagonist) decreased voiding frequency and volume in a mice model of loxoribine (a selective TLR-7 agonist)-induced IC, suggesting that TLR7 might represent a promising therapeutic target for IC/BPS [64]. Pretreatment with intravesically applied nanocrystalline silver, which is available as an impregnated wound dressing for treatment of burns, significantly decreased infiltration of mast cells, urine levels of histamine, and bladder explant TNFα release in PS/LPS-induced SD rat model of IC [65]. The use of pregabalin and gabapentin, neuromodulators that selectively bind to the alpha-2-delta (α2δ) subunits of voltage-gated Ca^2+^ channels, showed promising preclinical results in experimental models of IC/BPS. Pregabalin treatment decreased hyperalgesia and reduced inflammation by reducing proinflammatory cytokine production and inhibiting NF-κB activation [66], while systemic administration of gabapentin reduced cystitis-related pain and frequency of voiding [67]. Ceftriaxone, a β-lactam antibiotic, diminished visceral hypersensitivity in stress-induced IC rats [68], while neurokinin-1 receptor antagonist aprepitant, used to treat nausea, relieved pelvic pain, urinary symptoms, and bladder inflammation in mice with experimental autoimmune cystitis [69]. Given the substantial costs and time of new drug discovery and development, drug repurposing could represent an attractive option to treat IC/BPS patients, according to the promising results of preclinical research.

#### 3.2.3. Other Intravesical Therapies and Improved Drug Delivery Systems

Intravesically delivered therapeutic agents reduce systemic side-effects and improve treatment effects by maintaining local drug concentration [12]. Due to the significant disadvantages of intravesical drug delivery, such as low permeability of the urothelium and periodical voiding, which results in fast clearance of active substances with urine and subsequent need for repetitive catheterization, novel approaches, such as liposomes and hydrogels, are being developed [12]. For example, intravesical liposome instillation resulted in partially reversed shortening in inter-contraction interval in rat IC model [70]. Presumably, liposomes were able to form a protective film over damaged urothelium and prevent urinary irritants from acting on the afferent branch of the micturition reflex [70]. The superior effects of liposomes on reducing bladder hyperactivity in comparison to PPS and DMSO were later demonstrated by Tyagi et al. [71,72]. Lin et al. demonstrated that intravesical administration of heparin-loaded floating hydrogel extends the residence time of heparin and increases drug efficiency compared to direct intravesical administration of the drug in a rabbit model [73]. Additionally, a pilot study by Rappaport et al. later reported on the safety and efficacy of intravesical instillation of TC-3 hydrogel in combination with BTX-A. IC/BPS patients included in the study reported mild and temporary adverse effects with improvement in pain and bladder function persisting for 12 weeks [74]. Interestingly, Lee et al. developed an intravesical device for sustained drug delivery that can be implanted into and retrieved from the bladder non-surgically through a cystoscope. The device, combining a Nitinol wireframe and drug-loaded silicone tube, provided a sustained and localized lidocaine delivery while moving freely inside the bladder and preventing local irritation [75]. The method was recently upgraded by Xu et al. who used stereolithography (SLA) 3D printing for the fabrication of an intravesical drug delivery device. The SLA method enables the production of solid objects by polymerization of liquid resins under light irradiation, while the drugs can be incorporated into resin before printing. For the in vitro drug release study, lidocaine hydrochloride was added to elastic resin before printing, which provided a linear release of the drug from the solidified device across a 14-day period [76]. Another emerging drug delivery system includes mucoadhesive polymers, which enable greater bioavailability and solubility of poorly soluble drugs. Chitosan is a promising excipient for the development of such systems due to its positive charge and high mucoadhesive properties in acidic urine. Its favorable adhesion and prolonged drug residence time are being extensively researched, especially for the improvement of bladder cancer treatment options [77,78,79,80].

#### 3.2.4. Novel Emerging Therapeutic Options and Targets

##### Stem-Cell Therapy

Stem cells (SCs), including adult stem cells and pluripotent stem cells, such as embryonic stem cells and induced pluripotent stem cells, possess the abilities of self-renewal, proliferation, and differentiation into various cell types. The therapeutic effects of SCs have been extensively researched and preclinically trialed in many diseases, including IC/BPS [81]. To date, SCs of different origins have been tested in animal models of IC/BPS, including human umbilical cord blood, dental pulp, and adipose tissue. SCs injected intravesically or directly into bladder wall ameliorated bladder voiding dysfunction and inflammation, reduced nociceptive behavior, and decreased urothelial damage in rat IC models [82,83,84]. In addition, combination therapy of adipose tissue-derived SCs and oral PPS showed synergistic effects in normalizing bladder function and reducing inflammatory reaction [85]. Interestingly, urine-derived SCs (USCs) also possess the capacity for multipotent differentiation and can form multilayered tissue-like structures consisting of urothelium and smooth muscles in vivo [86]. Li et al. showed that USC treatment significantly ameliorated urodynamic, inflammatory, oxidative, and apoptotic changes in PS/LPS-induced IC in female SD rats, compared to untreated controls [87]. Recently, Chung et al. compared the therapeutic potency of mesenchymal SCs derived from different sources—urine, bone marrow, adipose tissue, and amniotic fluid, which showed no significant differences in the regeneration of urothelium and smooth muscle. However, urine-derived stem cells showed superior anti-inflammatory properties, compared to SCs derived from other sources [88].

Due to various limitations of using pluripotent embryonic SCs (ESCs) as a treatment option, such as ethical considerations, teratoma development, long-term possibility of carcinogenesis, and potential immunological rejection of transplanted SC [89], novel strategies of ESCs use are being intensively explored. In 2017, Kim et al. reported on the potential use of human ESC (hESC)-derived multipotent mesenchymal SCs (M-MSC) in IC/BPS, with evidence for long-term safety (6 months after transplantation). M-MSCs therapy significantly ameliorated defects in bladder voiding function, reduced visceral hypersensitivity, and expressed superior therapeutic potency compared to adult bone marrow-derived mesenchymal SCs given in the same doses [90]. The results were later confirmed by Lee et al. in the ketamine-induced chronic IC rat model [91]. Intravital imaging of transplanted hESC-derived M-MSCs in PS/LPS-induced rat IC model demonstrated migration of GFP-transfected M-MSCs from the injection site (serosa and muscle layer) to the damaged urothelium and lamina propria, followed by differentiation into various cell types, which correlated with improvement of IC/BPS symptoms [92].

More recently, Inoue et al. successfully generated differentiated urothelial cells (dUCs) from adult human dermal fibroblasts (aHDFs) using direct conversion technology, which enables conversion of differentiated somatic cell line without passing through a pluripotent state. This was achieved by transduction of a set of genes encoding transcriptional factors (e.g., FOXA1, TP63, MYCL, and KLF4) with crucial roles in the development of target cell lineage [93]. Following transduction, dUCs formed epithelial colonies and expressed urothelial specific proteins UP1b, UP2, CDH1, and Krt8/18 with an in vitro conversion rate of 25%. Moreover, transduced aHDFs, transplanted into a murine IC model, were able to convert into dUCs in vivo in the injured bladder urothelium and participate in tissue regeneration, after integrating into the inner bladder surface [94]. The preclinical studies mark SCs as favorable in IC/BPS treatment; however, no ongoing clinical trials on SC therapy of IC/BPS or overactive bladder are currently registered. Due to the several limitations of SC application, the results of animal studies should be carefully interpreted and critically evaluated before designing clinical trials.

##### Plant-Based Therapy

Plant-derived agents have recently gained a significant amount of interest in treating different inflammatory and chronic diseases due to their low level of toxicity, cost-effectiveness, and easy availability. In IC/BPS, the use of several medicinal plants has been evaluated in experimental models. For example, *Aster tataricus* extract and its main active component Shionone were shown to reduce bladder inflammation in IC rats and decrease pyroptosis in the SV-HUC1 urothelial cell line, by inhibiting the NLRP3–GSDMD pathway [95,96], while intravesical treatment with *Bletilla striata* extract solution was shown to attenuate visceral hypersensitivity and bladder overactivity in zymosan-induced-IC in SD rats [97]. Treatment with *Olea europaea* or *Juniperus procera* leaf extracts in SD rats with stress (water deprivation)-induced IC reduced bladder mast cell infiltration and levels of stress hormones [98], and *Houttuynia cordata* extract inhibited mast cell proliferation and activation, decreased the levels of proinflammatory cytokines IL6, IL8, and TNFα, and reduced inter-contraction intervals in SD rats with CYP-induced IC [99]. Epigallocatechin-3-gallate (EGCG), a major catechin found in green tea, showed an anti-inflammatory effect in stress-induced IC rats [100], as well as urothelial cells, isolated from bladders of IC/BPS patients via inhibition of phosphorylated NF-κB. EGCG also decreased the expression of purinergic receptors and showed antioxidative properties [101]. Adelmidrol, a diethanolamide derivative of natural azelaic acid, ameliorated CYP-induced bladder inflammation and pain by inhibiting the NF-κB pathway and inflammatory mediator levels, as well as reducing mechanical allodynia and NGF levels [102]. Administration of chlorogenic acid, a phenolic compound widely found in fruits and vegetables, significantly attenuated inflammation, by inhibiting the MAPK/NF-κB pathway and decreasing proinflammatory mediators IL6, IL1β, and TNFα in SD rats with CYP-induced IC [103]. More recently, Shih et al. discovered that curcumin administration mitigated bladder injury and reduced the levels of proinflammatory mediators in the combined PS/LPS mice IC model by downregulating the NLRP3 inflammasome/IL-1β-related TGF-β/Smad pathway [104].

##### Extracorporeal Shock Wave Therapy

Shock waves are sonic pulses that carry energy from an area of positive pressure to the area of negative pressure through a fluid medium (water or gel). Shock waves can be generated using different sources (electrohydraulic, piezoelectric, or electromagnetic type of generators), and they are characterized by the rapid rise (˂10 ns) and high peak pressure (up to 100 MPa) of short duration (˂10 µs) and a broad range of frequency spectrum (16 to 20 MHz) [105]. Shock wave therapy was originally used for the disintegration of nephroliths and uroliths, and it later provided immense value in the therapy of musculoskeletal disorders by promoting angiogenesis and tissue regeneration [106]. Chen et al. were the first to describe the beneficial effects of extracorporeal shock wave therapy (ECSWT) in a rat IC model. ECSWT, applied to the skin surface above the bladder or directly to exposed bladder dome, successfully attenuated bladder inflammation and oxidative stress, as well as contributed to the preservation of urothelial integrity in CYP-treated rats [107,108] and UPK3A-immunized mice [109]. The combination of ECSW and BTX-A has also produced advantageous results in overactive bladder and IC/BPS animal models. Transient urothelial permeability, following ECSWT, enables more efficient penetration of BTX-A from urine into the submucosa, without the need for an additional injection of the drug into the bladder wall [110,111]. ECSWT has already provided encouraging results in several randomized clinical trials for the treatment of chronic prostatitis-related pain in men [112,113] and also holds promise for translation to clinical use in IC/BPS patients [114].

##### Targeting Protease-Activated Receptors

One of the mechanisms, involved in inflammation and pain development in IC/BPS is an activation of protease-activated receptors (PAR), present in urothelial cells, nerve fibers, and bladder detrusor muscles. Many serine proteases, which can contribute to PAR activation, can be found in increased concentrations in the urine of IC/BPS patients, including tryptase and thrombin. In in vivo models, activation of PAR1 and PAR4 with specific agonists resulted in prominent bladder edema and PMN cell infiltration [115], while PAR1 receptor blockade improved urodynamic parameters in CYP-induced IC in rats [116]. Moreover, intravesical stimulation of PAR1 with thrombin can cause the urothelial release of macrophage migration inhibitory factor (MIF), a cytokine that acts as a pivotal mediator of acute and chronic inflammation [117]. Kouzoukas et al. discovered that increased abdominal mechanical hypersensitivity to von Frey stimulation, secondary to intravesical instillation of PAR1- and PAR4-activating peptides, was completely abrogated by MIF-antagonist ISO-1 pretreatment. However, PAR activation did not elicit any bladder inflammation, implying MIF involvement predominantly in the development of pain [118]. The pivotal role of MIF in CYP-induced bladder pain was later confirmed by Ma et al., using MIF knockout mice, in which the mechanical hypersensitivity could not be induced, in contrast to wild-type mice [119]. Along with MIF, activation of PAR4 also promotes high-mobility group box 1 protein (HMGB1) release in vitro and in vivo. HMGB1, when released from necrotic, damaged, or immune cells, such as macrophages, acts as one of the damage-associated molecular patterns (DAMPs) that can activate several receptors (i.e., receptor for advanced glycation end-products (RAGE) and Toll-like receptor 4 (TLR4)) and contribute to inflammatory and neuropathic pain [120]. The detrimental effects of HMGB1 in animal IC models were prevented by pretreatment with anti-HMGB1 neutralizing antibody [121,122] or HMGB1 antagonist glycyrrhizin [123], indicating that it could serve as a potential therapeutic target in IC/BPS.

##### Targeting Purinergic Receptors

Aggravated purinergic signaling has been shown to be one of the main factors in the development of IC/BPS-related bladder hypersensitivity; therefore, it is not surprising that purinergic P1 (activated by adenosine) and P2 (activated by ATP) receptors are rapidly emerging as potential drug targets. In a study by Hiramoto et al., the authors hypothesized that rapid urothelial ATP release, a common observation in IC/BPS patients, can be induced with CYP treatment in mice, followed by the activation of P2X4 and P2X7 receptors and subsequent HMBG1 release, resulting in bladder pain [124]. Administration of a specific P2X7 antagonist A-438079 was shown to markedly reduce CYP-induced nociceptive behavior and inflammation in a mouse model of hemorrhagic cystitis [125]. On the other hand, a study by Aronsson et al. later failed to reproduce the anti-inflammatory effects of P2 receptor blockade using suramin, a nonselective P2 antagonist in CYP-treated SD rats, supposedly due to species differences [126]. The activation of purinergic receptors can also be prevented by lowering the levels of ATP, released through pannexin channels. This was elegantly shown by Beckel et al. in LPS-treated SD rats. Concurrent administration of BB-FCF, a pannexin 1 channel inhibitor, decreased urothelial ATP release in basal and stretched conditions and completely reversed the LPS-induced decrease in inter-contraction interval [127].

In addition to P2 receptors, the blockade of adenosine receptor P1A1 with its antagonist DPCPX was shown to decrease mast cell infiltration in the detrusor of CYP-treated animals [126]. However, confounding results exist regarding the influence of activation or suppression of P1 receptors, specifically adenosine receptor A2a, on inflammatory response and bladder overactivity. Yang et al. reported that inhibition of adenosine A2a receptors with ZM241385, a selective A2a receptor antagonist, significantly alleviated bladder overactivity and hyperalgesia in CYP-treated animals by reducing the sensitivity of TRPV1 in DRG neurons [128], while Ko et al. showed that activation of adenosine A2a receptor with polydeoxyribonucleotide (PDRN), an A2a receptor agonist, improved voiding dysfunction and reduced inflammation and apoptosis [129]. Interestingly, anti-inflammatory effects of PDRN have previously been demonstrated in animal models of various diseases, such as ischemic colitis, gastric ulcers, and Achilles tendon injury [130,131,132].

##### Targeting Transient Receptor Potential Channels

Transient receptor potential (TRP) channels are nonselective ion channels, capable of responding to a variety of stimuli, such as mechanical pressure, changes in pH, heat, and different chemical compounds. Since a variety of chemical and physical stimuli can regulate their activation, TRP channels participate in numerous sensory or homeostatic processes, making them promising pharmacological targets. Their role in the development of lower urinary tract dysfunctions has been extensively investigated [133,134], and some of them have also been trialed as potential drug targets in animal IC models. The transcriptional and translational plasticity of TRPA1, TRPV1, and TRPV4 channels in CYP-induced acute and chronic IC models has been demonstrated by Merrill et al. [135], while selective blockade of TRPV4 channels resulted in improved bladder function [136,137]. Moreover, treatment with artemin-neutralizing antibody reversed CYP-induced hyperalgesia in female C57BL/6 mice by regulation of TRPA1 expression. Artemin, a neurotrophic factor released in inflammatory conditions, can sensitize afferent nerve endings in part through upregulation of expression or augmented function of TRPA1 and TRPV1 channels, contributing to bladder pain [138].

##### Targeting microRNA

MicroRNAs are short, approximately 22 nucleotide long noncoding RNA molecules that can post-transcriptionally regulate gene expression. Various microRNAs can serve as biomarkers of different lower urinary tract diseases, such as bladder cancer [139], bladder outlet obstruction [140], overactive bladder [141], and IC/BPS [142,143], and they have also been recognized as emerging therapeutic targets. In the present review, we identified two studies trialing microRNAs as potential drug targets in animal models of IC/BPS. Song et al. showed that inhibition of miR-132 reduced bladder inflammation and detrusor fibrosis, as well as improved urodynamic parameters in a PS/LPS-induced rat model of IC via regulation of JAK/STAT signaling pathway [144]. Similarly, the application of miR-495-mimic in a rat model of acute IC resulted in JAK3 downregulation and subsequent inhibition of inflammatory response and bladder fibrosis [145].

##### Activation of the Cannabinoid System

Synthetic and semisynthetic cannabinoids that lack psychotropic effects have gained much interest recently regarding the treatment of chronic inflammatory disorders and pain, including IC/BPS, due to their antiproliferative, anti-inflammatory, and analgesic effects [146]. The effects of cannabinoids are mediated primarily through cannabinoid receptors CB1 and CB2 that have been found to be present in human and rodent bladder urothelium and detrusor smooth muscle cells [147]. In line with this, Hayn et al. observed that ajulemic acid, a mixed CB1/CB2 receptor agonist, inhibited CGRP release ex vivo in capsaicin- and ATP-stimulated whole rat bladders [148]. Tambaro et al. later showed that administration of JWH015, a selective CB2 agonist, significantly reduced leukocyte infiltration and proinflammatory cytokines in bladder interstitium of CD1 mice [149]. Treatment with another selective CB2 agonist GP1a also decreased severity of edema in acrolein-induced cystitis, inhibited mechanical sensitivity, and decreased bladder urinary frequency that may be mediated by inhibition of ERK1/2 pathway [150,151]. Recently, Liu et al. showed that JWH-133, a selective CB2 agonist completely inhibited mechanical hyperalgesia in CYP-induced mice IC, as well as ameliorated bladder inflammation and oxidative stress. The protective effects of CB2 activation against CYP-induced IC could be mediated by autophagy activation since CB2-induced AMPK activation inhibited mTOR signaling, which subsequently activated autophagy [152]. Berger et al. administered beta-caryophyllene (BCP), which is present in cannabis and activates CB2, to mice with LPS-induced IC. BCP reduced bladder inflammation and improved bladder capillary perfusion with comparable effects to the selective CB2 agonist, HU308 [153]. Palmitoylethanolamide, an endogenous lipid chemically related to the endocannabinoid anandamide, which, in addition to PARα, activates CB1 and CB, was found to be able to attenuate pain behavior, voids, and bladder gross damage in a CYP-induced IC mice model [154]. Pharmacological evidence suggests that, in addition to targeting the canonical cannabinoid receptors (e.g., CB1 and CB2), the cannabinoids can also modulate some TRP channels (TRPV1–4, TRPA1, and TRPM8) [155], thus providing a promising multitarget approach for the treatment of IC/BPS. Despite their great potential, however, there are currently no ongoing clinical trials testing their use in patients with IC/BPS.

### 3.3. Methodological Quality and Risk of Bias

To evaluate the methodological quality of studies included in the present review, we defined a 12-point checklist based on published ARRIVE guidelines describing minimal information required in scientific publications including animal models [26]. The median number of quality items scored was six out of a possible 12 (q25–q75: 2–7) (Table S3, Supplementary Materials). All publications using in vivo experimental models (*n* = 88) reported the species and strain of the animals included in their study, as well as detailed description of experimental procedures, such as drug formulation, dose, site, and route of administration. Surprisingly, 86/88 (99%) and only 41/87 (47%), 53/87 (61%), and 8/87 (9%) studies included information regarding the sex, exact age, weight, and detailed description of housing and husbandry of the animals, respectively. The total number of animals used was indicated in 70 (80%), while the exact number of experimental (treated and control) groups was reported in 63 (74%) studies. Measures to avoid bias were infrequently reported, with 18 (21%) publications reporting on random allocation of the animals to treatment groups, and none of the studies describing the method of randomization. Blinded assessment of outcome was included in 18 studies (21%), blinding of the investigator/caretaker was reported in four (5%) of the included studies, and one study (1%) described the method used to calculate the sample size (e.g., number of animals per group), a determination required to avoid false outcomes. Our review suggests that the prevalence of measures introduced to reduce the risk of bias and detailed experimental reporting should be substantially increased to improve the reproducibility and interpretability of the studies, as well as to avoid potential false-positive results and overestimates of treatment effects.

## 4. Conclusions

Currently, the number of in vitro studies on IC/BPS is very limited, and most of them use urothelial cell lines that are transformed and do not form tight monolayers similar to normal urothelium. Although the in vitro experimental design does not reflect the complexity of the in vivo condition, these studies can lead to a better understanding of the pathology of IC/BPS at the cellular and molecular level. Unraveling the exact relationship between altered urothelial, neuronal, smooth muscle, and/or immune signaling, and the clinical symptoms/signs in IC/PBS will be of outmost importance for understanding the disease process and may help to identify promising targets for future treatment.

Preclinical studies involving animal models remain imperative in studies of etiology and pathophysiology of IC/BPS, as well as novel drug target discovery, and they can help to inform the design of clinical trials. However, adequate experimental design and study quality are important factors for successful implementation into a clinical setting. Our results show that the methodological quality of animal studies could be considerably improved and measures to avoid bias should be implemented and adequately reported. Future studies should incorporate a more standardized and rigorous approach for animal modeling in order to increase the value of preclinical research and the translational potential of experimentally evaluated therapies and therapeutic targets for IC/BPS. Despite several limitations of preclinical experimental models, novel conclusions are drawn almost daily, increasing the insight into the complex mechanisms of IC/BPS development, and several novel treatment options are emerging.

Currently, there is no definite therapeutic modality available and recommended that would be consistently successful in all IC/BPS patients. Most patients are treated on the basis of a “trial and error” approach and need to undergo a series of different combinations of therapies, facing potential severe adverse events. Intravesical application of GAG replenishment therapy, DMSO, and BTX-A ensures maximum delivery of active drug ingredients into the bladder; however, repeated catheterizations are required, causing frequent urinary tract infections. A combination of intravesically delivered therapeutic agents with recently improved drug delivery systems, such as liposomes, hydrogels, and biodegradable polymers and/or a simplified approach of 3D printing to manufacture novel indwelling bladder devices will likely contribute to sustained therapeutic effect without the need for repeated instillation. This will diminish systemic side-effects and enable drug delivery over an extended period of time, ensuring a long-lasting therapeutic effect.

As IC/BPS is a multifactorial disease with several proposed mechanisms of pathobiology, it is anticipated that a multitarget therapeutic approach will be required to achieve long-term efficacy. Stem cells, ECSWT, and activation of the cannabinoid system, which modulate several aspects of the diseases, including the inflammatory processes, central sensitization, pain, and tissue repair, have been proven to be effective in several preclinical studies and have a great translational potential. However, current clinical studies are limited to case reports, and large, multicenter, long-term, randomized clinical trials are warranted to elucidate their efficiency and safety in patients with IC/BPS.

Furthermore, phenotyping and stratifying patients into subgroups as a function of clinical signs and bladder histological findings will be particularly important for selection of patients most suitable for a specific therapeutic option. Due to the extremely complex and heterogeneous pathological backgrounds of IC/BPS patients, the currently used “one-size-fits-all” medicine should be replaced with a more personalized approach, also takinginto account the variability in genes, environment, and lifestyle of a particular patient.

## Data Availability

No new data were created or analyzed in this study. Data sharing is not applicable to this article.

## Supplementary Materials

The following are available online at https://www.mdpi.com/article/10.3390/biomedicines9080865/s1, Table S1. In vitro and ex vivo experimental models of IC/BPS evaluating different therapeutic options; Table S2. In vivo experimental models of IC/BPS evaluating different therapeutic options; Table S3. Methodological quality and reported measures undertaken to avoid bias.

## Abbreviations

A2	adenosine receptor
Ab	antibody
ACh	acetylcholine
ACTH	adrenocorticotropic hormone
AJA	ajulemic acid
APF	antiproliferative factor
ASC	apoptosis-associated speck-like protein
A7R5	smooth muscle cell line
AYPGKF-NH2	PAR4 agonist
BCP	beta-caryophyllene
BTX-A	botulinum toxin A
CAT	catalase
CB	cannabinoid receptor
CBX	carbenexolone
CD	cluster of differentiation
CGRP	calcitonin gene-related peptide
CRH	corticotropin-releasing hormone
CS	chondroitin sulfate
CYP	cyclophosphamide
DMSO	dimethyl sulfoxide
DRG	dorsal root ganglia
ECSWT	extracorporeal shock wave therapy
EGCG	epigallocatechin gallate
eNOS	endothelial nitric oxide synthase
EP	prostaglandin E2 receptor subtype
ERK	extracellular signal-regulated kinase
FcεRIα	high-affinity IgE receptor
FTLK	transcriptional factors FOXA1, TP63, MYCL, and KLF4
GAG	glycosaminoglycan
GM-0111	modified GAG
GSDMD	gasdermin D
GSH	glutathione
HA	hyaluronic acid
H-BLAK	primary human bladder cell line
h-ESC	human embryonic stem cells
HMGB1	high mobility group box 1
HO-1	heme oxygenase-1
H_2_O_2_	hydrogen peroxide
HTB4, HRB2	human urothelial cells
ICAM	intercellular adhesion molecule
IC/BPS	interstitial cystitis/bladder pain syndrome
ICI	intercontraction interval
IFN-γ	interferon gamma
IκBα	inhibitor of NF-κB
IL	interleukin
iNOS	inducible nitric oxide synthase
JAK	janus kinase
KC	keratinocytes-derived chemokine
LDH	lactate dehydrogenase
LL37	antimicrobial peptide
LPS	lipopolysaccharide
MAPK	mitogen-activated protein kinase
MCP-1	monocyte chemoattractant protein-1
MDA	malondialdehyde
MIF	macrophage migration inhibitory factor
miR	microRNA
MMP9	matrix metalloproteinase 9
M-MSC	multipotent mesenchymal stem cells
MPO	myeloperoxidase
MSC	mesenchymal stem cells
NF-κB	nuclear factor kappa B
NGF	nerve growth factor
NK1R	neurokinin 1 receptor
NLRP3	NLR family pyrin domain-containing 3
NMDAR	*N*-methyl-d-aspartate receptor
NOX	NAPDH oxidase
NQO-1	NADPH quinine oxidoreductase
NRK-52E	renal tubular epithelial cell line
NVC	non-voiding contraction
OVX	ovariectomized
P1, P2	purinoceptors
P2X	purinergic receptors
p38	mitogen-activated protein kinase
p65	NF-κB subunit
p-AKT	protein kinase B, phosphorylated
PAR	proteinase-activated receptor
PGE2	prostaglandin 2
PMN	polymorphonuclear cells
p-mTOR	mechanistic target of rapamycin, phosphorylated
POMC	pro-opiomelanocortin
PPAR	peroxisome proliferator-activated receptor
PPS	pentosan polysulfate sodium
PS	protamine sulfate
PTX3	pentraxin 3
RANTES	chemokine ligand 5
RhoA	Ras homolog gene family, member A
rhsTM	recombinant human soluble thrombomodulin
ROCK	Rho-associated protein kinase; R
OS	reactive oxygen species;
RT112	3D human bladder epithelium preparation
SAGE	semi-synthetic glycosaminoglycan ethers
SC	stem cells
SD rats	Sprague-Dawley rats
sGC	soluble guanylyl cyclase
MAPK	mitogen-activated protein kinase
MCP-1	monocyte chemoattractant protein-1
MDA	malondialdehyde
SMAD	proteins for signal transduction of the transforming growth factor beta superfamily
TRPV	transient receptor potential channel, vanilloid subgroup
UPK	uroplakin
VEGF	vascular endothelial growth factor
ZO-1	tight junction protein 1
