# Supplementary material for: A Systematic Review of Therapeutic Approaches Used in Experimental Models of Interstitial Cystitis/Bladder Pain Syndrome
Source: Biomedicines. 2021 Jul 22;9(8):865. doi: 10.3390/biomedicines9080865 (PMC8389661; doi:10.3390/biomedicines9080865)
Supplement: Supplementary file 1 [file biomedicines-09-00865-s001.zip › biomedicines-1313742-supplementary.pdf]

Supplementary table S1: ***In vitro*** **and** ***ex vivo*** **experimental models of IC/BPS evaluating different therapeutic options.**

REF.	CELL TYPE	STIMULATION	TREATMENT	MAIN FINDINGS & OUTCOME
**AGENTS RECOMMENDED BY AUA GUIDELINES TO TREAT IC/BPS**				
Rooney et al., 2015 [1]	HTB4	TNFα (10 ng/ml), 24h PS (100 ng/ml), 30 min	HA (0.4 mg/ml), 24h	decreased IL6, IL8, transepithelial permeability, increased sulfated GAG
Stellavato et al., 2019 [2]	H-BLAK, RT112	TNFα (10 ng/ml)	HA (0.6 and 1% w/v), CS (0.6 and 2% w/v), HA + CS (1.6% +2% w/v), 4-72h	reduced IL6, IL8, NFκB, increased ZO-1, cell proliferation
Rozenberrg et al., 2019 [3]	Porcine urothelial cells	PS (10 mg/ml), 4h	CS (0.2% vol/vol), 7h and 24h	accelerated barrier recovery time
Rooney et al., 2020 [4]	HTB4	TNFα (10 ng/ml), 3-24h/ PS (100 ng/ml)/H_2O_2 (1%), 1h	Cystistat (0.8 mg/ml HA), iAluRil (16 mg/ml HA, 20 mg/ml CS), Hyacyst (0.8 mg/ml HA), 2h	increased sulfated GAG, reduced COX-1
Rooney et al., 2020 [5]	HTB2, T84	TNFα (100 ng/ml)/ PS (10 mg/ml)/H_2O_2 (1%), 1h	cHA:HA (1 and 3 mg/ml, 1:1, 1:10 ratio), 2-72h	cHA:HA (1:1) decreased permeability, no change in IL6, IL8, MCP1; cHA:HA (1:10) increased IL6 and MCP1, no effect on permeability
Lee et al., 2013 [6]	human urothelial cells	LL37 (0.3-25 μM), 15 min	GM-0111 (3 μm), 30 min pretreatment	reduced apoptosis and ATP release
Rajasekaran, et al. 2006 [7]	Rat urothelial cells	toxic factor (2 mg/ml), PS (2 mg/ml), overnight	PPS (2 mg/ml), 2h premixed with toxic factor	decreased cytotoxicity
Melchior et al., 2003 [8]	Rat detrusor muscle strips	ACh (5 nM), KCl (120 mM)	DMSO (25-50%), 7 min pretreatment	40% DMSO completely blocked induced contraction, 30% DMSO decreased compliance
Rapp et al., 2006 [9]	Whole rat bladders	capsaicin (30 nM), ATP (10 μM)	BTX-A (50 μM), 6h pretreatment	decreased CGRP
Lucioni et al., 2007 [10]	Whole rat bladders	HCl (0.4 M), 10 s	BTX-A (10 U), 1h	decreased CGRP and substance P
**AGENTS APPROVED TO TREAT OTHER DISEASES**				
Grundy et al., 2020 [11]	Whole mouse bladders	histamine (300 μM), 5-15 min	pyrilamine (100 μM), 5-15 min	Reduced mechanical hypersensitivity
Zhang et al., 2017 [12]	Mouse urothelial cells, NRK-52E	acrolein (100 μM), TRPV4 agonist [4α-PDD12] (10 μM), 6-12h	CBX (10 μM), 6-12h	reduced cell injury, intracellular Ca^{2+}, LDH, inhibited P38 phosphorylation
Boudieu et al., 2019 [13]	Mouse peritoneal exudate cells	LPS (100 ng/ml), 10-90 min, LPS (1 μg/ml), 2 min	pregabalin (11.3 μM), 10-90 min or 15 min pretreatment	reduced α2δ-1, phospho-p65, IL6 and intracellular Ca^{2+}

**EMERGING THERAPEUTIC OPTIONS**				
Xie et al., 2018 [14]	SV-HUC-1	TNFα (10 ng/ml), 24-72h	human umbilical cord MSC, coculture, 24-72h	increased proliferation, p-AKT and p-mTOR, decreased apoptosis, cleaved caspase-3, IL1β, IL6, and TNFα
Wang et al., 2020 [15]	SV-HUC-1	LPS (10 ug/ml), ATP (2.5 mM), 12h	*Aster tataricus* extract (25-100 ug/ml), 2h pretreatment	Decreased cell damage, caspase-1 p20, pro-caspase-1, NLRP3, GSDMD and GSDMD-N
Wang et al., 2021 [16]	SV-HUC-1	LPS (10 ug/ml), ATP (2.5 mM), 12h	Shionone (2.5-10 μg/ml), 2h pretreatment	increased cell viability, decreased NLRP3, caspase-1, ASC, IL1β, GSDMD, NFκB, and GSGMD-N
Liu et al., 2013 [17]	human urothelial cells	stretch cycles, 24-96h	EGCG (0.1-1% vol/vol), 24-96h	decreased ATP release
Chen et al., 2014 [18]	A7R5	menadione (25 μm), 30 min LPS (500 ng/ml), 3h	ECSWT (200 shots; 0.12 mJ/mm^2), 3h pretreatment	reduced TNFα, IL1β, IL6, MMP9, NFkB, NOX1, NOX2, and oxidative index (protein carbonyls)
Monjotin et al., 2016 [19]	Whole rat bladders	thrombin (3 U/ml)/PAR1 agonist [TFLLR-NH2] (1-100 μm)	PAR1 agonist [F16357] (10-30 μM), 30 min pretreatment	suppressed the maximal contraction and reduced the rhythmic instability
Yang et al., 2021 [20]	L6-S1 rat DRG neurons	capsaicin (1 μM), NA	adenosine A_{2a} receptor antagonist [ZM241385] (10 μM), 2 min pretreatment	inhibited Ca^{2+} influx
Hayn et al., 2008 [21]	Whole rat bladders	capsaicin (30 nM), ATP (10 μM), 10 min	CB1/CB2 agonist AJA (IP-751) (75 nm), 1h pretreatment	reduced CGRP
**OTHER THERAPEUTIC AGENTS AND TARGETS**				
Keay et al., 2011 [22]	Human urothelial cells	synthetic as-APF (125 nM), 48h (normal cells)	D-proline as-APF (2.5 μM), D-pipecolic acid as-APF (2.5 μM), 48h-30 days (normal and IC/BPS cells)	attenuated as-APF antiproliferative activity, paracellular permeability, stimulated cell proliferation, increased ZO-1, occludin, and claudins-1, 4, 8, 12
Coelho et al., 2014 [23]	Rat urothelial cells	NGF (100 ng/mL), 15 min, capsaicin (0.5 μm), 15-20 min	TrkA antagonist [GW441756] (500 nM), 30 min pretreatment	decreased TRPV1 and ATP

Supplementary table S2: ***In vivo*** **experimental models of IC/BPS evaluating different therapeutic options.**

REF.	ANIMALS	IC INDUCTION	TREATMENT	MAIN FIDINGS & OUTCOME
**AGENTS RECOMMENDED BY AUA GUIDELINES TO TREAT IC/BPS**				
**Acute IC models**				
Hauser et al., 2009 [24]	BALB/c mice (female), SD rats (female), 300g	HCl (10 mM), intravesically, 10 min	CS (20 mg/ml), intravesically, 30 min	restored permeability barrier
Yeh et al., 2010 [25]	Wistar rats (female), 220-250g, n=20	H_2O_2 (0.3%/1.5%), intravesically	HA (0.8 mg/ml), intravesically, 30 min	attenuated bladder hyperactivity, decreased ATP and ACh
Greenwood et al., 2018 [26]	SD rats (female), OVX, 220-250g	PS (1 mg/ml), intravesically, 10 min	lubricin (1.2 mg/ml), intravesically	decreased permeability
Jensen et al., 2019 [27]	C57BL/6 mice (female), 8-10 weeks, n=40	LL-37 (320 μM), intravesically, 1h	SAGE GM-0111 (10 mg/ml), intravesically, 5 min	reduced pain and inflammation
Towner et al., 2021 [28]	SD rats (female), OVX; URO-MCP-1 transgenic mice (female)	Rats: PS (1 mg/ml), intravesically, 10 min; Mice: LPS (10 μg/ml), intravesically, 1h	SuperGAG (20 mg/ml), intravesically	restored TEER and abrogated visceral pain in rats; restored permeability in mice
Lee et al., 2013 [6]	C57BL/6NCrl mice (female), 8-11 weeks	LL-37 (250 μl), intravesically, 1h	GM-0111 (150 μl), intravesically, 1h pretreatment	reduced weight loss, bladder edema, IL6, PTX3, MPO, serum amyloid P
Engles et al., 2013 [29]	SD rats, 300g	HCl (10 mM), intravesically, 10 min	CS (20 mg/ml), intravesically, 20 min	reduced edema, mast cell, and neutrophil count
Ottamasathien et al., 2011 [30]	C57BL/6 mice (female), 8-12 weeks, n=24	LL-37 (320 μM), intravesically, 45 min	SAGE GM-1111 (10 mg/ml), intravesically, 45 min pre- or post-treatment	decreased PMN count, MPO
Rajasekaran et al., 2006 [7]	SD rats (male), 325-350g, adult, n=18	human urine toxic factor (15 mg/ml), KCl (29.8 mg/ml), intravesically, 30 min	PS (10 mg/ml), premixed with stimul. agent, intravesically, 30 min	lowered number of NVC
Soler et al., 2008 [31]	Wistar rats (female), 180-200g, n=108	PS (5%), intravesically, 30 min	DMSO (50%), intravesically, 30 min	reduced edema PMN count, urine-excreted HA
**Chronic IC models**				
Smith et al., 2005 [32]	SD rats (female), 200-250g, n=24	PS (1%), intravesically, 30 min on day 0; CYP (150 mg/kg) i.p. every 3rd day - total of 3 injections	BTX-A (20 U/ml), intravesically, 30 min, pretreatment	reduced bladder hyperactivity and ATP release
Chuang et al., 2009 [33]	SD rats (female), 220-270g, n=60	CYP (75 mg/kg), i.p. every 3rd day on days 1, 4, 7	BTX-A (20 U/ml), intravesically, 1h on day 2	decreased bladder hyperactivity, inflammatory reaction, COX-2, EP4

**Acute and chronic IC models**				
Kim et al., 2011 [34]	URO-OVA/OT-1 transgenic mice (female), 8-10 weeks	Acute: activated OT-1 splenocytes (1×10^6), i.v., chronic: spontaneous development of IC at 10 weeks	DMSO (50%), intravesically, 1h, Acute: 3x after IC induction (days 1, 4, and 7) Chronic: 1x/week, 10 weeks	reduced cellular infiltration, edema, prevented triggering of acute cystitis after transfer of OT-1 splenocytes, reduced IFNγ, MCP1, NGF, TNFα, IL6
**AGENTS APPROVED TO TREAT OTHER DISEASES**				
**Acute IC models**				
Boucher et al., 2008 [35]	SD rats (female), 175-200g, n=37	PS (10 mg/ml), intravesically, 30 min, LPS (2 mg/ml), intravesically, 45 min	nanocrystalline silver (1%), intravesically, 20 min pretreatment	decreased lymphocyte and mast cell count, histamine and TNFα
Funahashi et al., 2014 [36]	SD rats (female), 230-260g, n=18	HCl (0.1 M), intravesically, 1 min	rebamipide (1/10 mM), intravesically, 1h, on days 1 and 4	decreased PMN cell count, edema, TNFα, IL1β, IL-6, UPK3A, urothelial permeability, nociceptive behavior, and normalized voiding parameters
Zhang et al., 2017 [12]	C57BL/6J mice (female), 20-25g, 10-12 weeks, n=56	CYP (150 mg/kg), i.p.	carbenoxolone (50 mg/kg), i.p., 1x/12h, 36h, pretreatment	normalized bladder function, reduced bladder edema, injury, COX2 and iNOS
Boudieu et al., 2019 [13]	C57BL/6J mice (male), 20-24g, n=32	CYP (150 mg/kg), i.p.	pregabalin (30 mg/kg), s.c.	reduced hyperactivity to von Frey filament, edema, MPO activity, IL6, KC, TNFα, α2δ1 receptor subunit, inhibited NF-κB and ERK1/2
Ichihara et al., 2017 [37]	C57BL/6N mice (female), 19.7-19.9g	loxribine (4.5 M), intravesically, 1h	hydroxychloroquine (100 mg/kg), p.o., 1x/4h, 12h, 1h pretreatment	decreased voiding frequency, increased voided volume, and ICI
Centinel et al., 2010 [38]	Wistar albino rats (female), 180-200g, adult, n=32	PS (5 mg/ml), intravesically, 2x in 24h	montelukast (10 mg/kg), i.p., 2x/day, 3 days	decreased inflammatory cell infiltration, mast cell number, MDA, increased GSH
**Chronic IC models**				
Holschneider et al., 2020 [39]	Wistar-Kyoto rats (female), adult, n=20	water avoidance stress, 1h/day, 10 days	ceftriaxone (200 mg/kg), i.p., 1h pretreatment	decreased visceral hypersensitivity and stress-related cerebral activations within the supraspinal micturition circuit
Bicer et al., 2015 [40]	BALB/cJ mice (female), 8-10 weeks, n=30	immunization with UPK3A 65-84 (1μg/μl)	cromolyn sodium/cetirizine/ranitidine (10 mg/kg), p.o., 1x/2h, 6h	reduced mechanical hyperalgesia
Liu et al., 2019 [41]	C57BL/6 mice (female), 6-8 weeks, n=30	emulsion of homogenized mice bladders (400 μl), s.c., 2 weeks intervals	aprepitant (1.2 mg/kg), p.o., 1x/day, 4 weeks	relieved pelvic pain, improved voiding behavior, reduced bladder edema, leukocyte infiltration, mast cells, ICAM-1
Yoshizumi et al., 2021 [42]	SD rats (female), 200-300g, adult	LPS (1 mg/ml), intravesically, 30 min, 1x/day, 4 days	gabapentin (30-300 mg/kg), p.o./i.v.	reduced pain-related behavior, prolonged ICI

**OTHER INTRAVESICAL THERAPY AND IMPROVED DRUG DELIVERY SYSTEMS**				
**Acute IC models**				
Chuang et al., 2003 [43]	SD rats (female), 250-300g, n=14	acetic acid (0.3%), intravesically	POMC cDNA (100 μg/0.1 ml), direct injection into bladder wall, 3 days pretreatment	reduced bladder hyperactivity
Gonzales et al., 2005 [44]	C57BL6 mice (female), 10-12 weeks	LPS (100 μg/ml), intravesically, 45 min	anti-inflammatory synthetic decapeptide [RDP58] (1 mg/ml), intravesically, 30 min	decreased inflammation (PMN infiltrates, edema), reduced TNF-α, NGF, and SP production
Tyagi et al., 2008 [45]	SD rats (female), 200-250g	PS (10 mg/ml), KCl (500 M), intravesically, 1h	liposomes (2 mg/ml in 500 nM KCl), intravesically, 2h	reduced bladder contraction frequency
Fraser et al., 2003 [46]	SD rats (female), 250-300g, n=34	PS (10 mg/ml)/KCl (500 mM)/acetic acid (0.1%), intravesically, 1h	liposomes (2 mg/ml in 500 mM KCl), intravesically, 1 or 2 h, pre- or post-treatment	reduced bladder hyperactivity
Tyagi et al., 2009 [47]	SD rats (female), 250-300g, n=24	PS (10 mg/ml), KCl (500 mM), intravesically, 1 h	liposomes (2 mg/ml in 500 mM KCl), intravesically, 2h	increased ICI
Konkol et al., 2016 [48]	SD rats (female), 10-11 weeks, n=52	HCl (0.4 M), intravesically, 90 s	cis-UCA (2%), intravesically, 1h, 2x/day, 3 days	decreased urinary frequency and edema, increased voided volume
**STEM CELL THERAPY**				
**Acute IC models**				
Song et al., 2015 [49]	SD rats (female), 10 weeks, n=45	HCl (0.1 M), intravesically, 10 min	umbilical cord blood-derived MSCs ($1x10^6$ cells) into submucosal layer of bladder wall	increased ICI and regeneration, reduced urothelial denudation, mast cell infiltration, angiogenesis, tissue fibrosis
Hirose et al., 2016 [50]	F344/NSIc rats (female), 120-140g, n=60	HCl (0.1 mol/L), intravesically, 1 min	dental pulp SCs ($2x10^6$ cells), intravesically	reduced edema, submucosal hemorrhage, tissue fibrosis, mast cell count, apoptosis, MPO activity, and nociceptive behavior, increased ICI
Kim et al., 2017 [51]	SD rats (female), 10 weeks, n=45	HCl (0.1 M) intravesically, 10 min	hESC-derived M-MSC ($2.5x10^5$ cells) into submucosal layer of bladder wall	reduced non-voiding contractions, urothelial denudation, mast cell infiltration, fibrosis, apoptosis, increased regeneration
Li et al., 2017 [52]	SD rats (female), 200-230g, n=60	PS (10 mg/ml) intravesically, 30 min, LPS (2 mg/ml) intravesically, 45 min	urine-derived SC ($1.2x 10^6$ cells), i.v.	reduced urothelial ulceration, edema, hemorrhage, inflammatory cell infiltration, mast cell count, IL6, TNFα, NF-κB, caspase 3, Bax, HO-1, and NQO-1, increased Bcl-2, micturition function

**Chronic IC models**				
Xie et al., 2018 [14]	SD rats (female), 200-250g, n=30	CYP (75 mg/kg), i.p., days 1, 3 and 5	Umiblical cord-derived MSCs ($1x10^6$ cells), injected through tail vein	increased bladder voiding function, decreased submucosal edema, hemorrhage, mast cell infiltration, IL-1β, IL6, TNFα
Kim et al., 2020 [53]	SD rats (female), 8 weeks, n=24	UPK3A (200 μg), s.c.	adipose tissue-derived MSC ($1x10^6$ cells) into bladder wall in combination with PPS (25 mg/kg/day), p.o.	reduced pain score, mast cell count, fibrosis, TNFα, IFNγ, MCP, IL6, TLR2, TLR11, prolonged ICI
Inoue et al., 2019 [54]	NOG/SCID mice (female), 6-8 weeks	anti-asialo GM1 antibody (100 mg), i.p. and H_2O_2 (1.5%), intravesically, 1h, dispase II (12.000 PU), intravesically, 1h	adult human dermal fibroblasts transduced with FTLK ($3x10^6$ cells/100 μl), intravesically, 3h	*in vivo* conversion into urothelial cells and regeneration of damaged bladder tissue
Ryu et al., 2018 [55]	SD rats (female), 10 weeks	PS (10 mg), intravesically, 45 min, LPS (750 μg), intravesically, 30 min, 1x/week, 5 weeks	hESC-derived M-MSC ($1x10^6$ cells) into bladder wall	improved non-voiding contractions, reduced urothelial denudation, mast cell infiltration, and apoptosis
Furuta et al., 2018 [56]	F344 rats (female), 160-200g, n=90	HCl (0.1 N), intravesically, 4 min, 1x/week, 2 weeks	adipose tissue-derived MSC ($1x10^6$ cells) into bladder wall	decreased nociceptive behavior, mast cells, collagen fibers, TNFα, TGFβ, normalized cystometric parameters
Chung et al., 2019 [57]	SD rats (female), 8 weeks, n=25	UPK2 (200 μg), s.c.	amniotic fluid-derived/ adipose tissue-derived/bone marrow-derived/urine-derived SC ($1x10^5$ cells), into bladder wall/tail vein/ intravesically	increased ICI, reduced voiding frequency, mast cell and T-cell infiltration, MPO, IL1β, IL6, IL17, TLR4, TLR5, TLR11, UPK-3, CD31, ZO-1
Lee et al., 2018 [58]	SD rats (female), 10 weeks, n=40	ketamine hydrochloride (25 mg/kg), i.v./i.p., 2x/week, 12 weeks	hESC-derived M-MSC ($0.25x/0.5x/1x10^6$ cells) into bladder wall	normalized cystometric parameters, reduced mast cell infiltration apoptosis, tissue fibrosis, TGFβ, SMAD, increased IL-10
**PLANT-BASED THERAPY**				
**Acute IC models**				
Wang et al., 2020 [15]	SD rats (female), 180-200g, n=40	CYP (150 mg/kg), i.p., 9 days	*Aster tataricus* extract (1.2 and 2.4 g/kg), gavage, 9 days	reduced edema, hemorrhage, inflammation index, NLRP3, caspase-1, ASC, IL-1β, GSDMD, and GSGMD-N
Wang et al., 2021 [16]	SD rats (female), 180-200g, n=40	CYP (150 mg/kg), i.p., 3 days	Shionone (50 and 100 mg/kg), gavage, 6 days pretreatment	reduced edema, hemorrhage, NLRP3, caspase-1, ASC, IL-1β, NF-κB, GSDMD, and GSGMD-N

Nassir et al., 2019 [59]	SD rats (female), 200-250g, 4 months, n=72	water-immersion restraint stress, 30 min	*Olea europea*/*Juniperus procera* extracts (250 and 500 mg/kg), p.o.; 14 days pretreatment	reduced mast cell infiltration, stress hormones (CRH and ACTH)
**Chronic IC models**				
Bazi et al., 2012 [60]	SD rats (female), 190-225g, n=20	water avoidance stress, 2h/day, 7 days	EGCG (1 mg/kg), i.p., 7 days pretreatment	decreased micturition frequency, inflammation score, total and degranulated mast cell counts
Li et al., 2020 [61]	SD rats (female), 200-250g, n=30	CYP (75 mg/kg), i.p, 1x/3 days, 10 days	*Houttuynia cordata* extract (2 mg/ml), intravesically, 1x/day, 1 week	decreased nociceptive behavior, inflammatory grade, mast cell number, IL6, IL8, TNFα
Luo et al., 2020 [62]	SD rats (female), 210-230g, n=80	CYP (75 mg/kg), i.p., 1x/3 days, 12 days	chlorogenic acid (100 mg/kg), i.p., 1x/day, 10 days	reduced IL6, IL1β, TNFα, apoptosis, caspase3, Bax, inhibited MAPK/NF-κB pathway, increased recovery of urinary function
Shih et al., 2021 [63]	BALB/c mice (female), 22-26g, 8 weeks, n=18	PS (1.5 mg), intravesically, 30 min, LPS (150 μg), intravesically, 30 min, 2x/week, 5 weeks	curcumin (100 mg/kg), p.o., 5x/week, 2 weeks	decreased bladder injury and micturition, tissue fibrosis, NLRP3, ASC, IL1β, TGFβ1, p-Smad2, p-Smad3
Liu et al., 2021 [64]	SD rats (female), 250-300g, 59th–65th day, n=30	zymosan (1%), intravesically, 30 min, 3x/2 weeks	*Bletilla striata* extract (25%), intravesically, 30 min, 1x/day, 7 days	decreased micturition interval and pain
**Acute and chronic IC models**				
Ostardo et al., 2018 [65]	Acute: SD rats (female), 200-250g, n=30<br>Chronic: CD1 mice (female), 25-30g, adult, n=30	Acute: CYP (200 mg/kg), i.p., 4h<br>Chronic: CYP (100 mg/kg), i.p., 1x/day, 5 days	Vessilen (2% adelmidrol + 0.1% sodium hyaluronate), intravesically, chronic: 1x/day, 1 week	reduced bladder inflammation, pain, mechanical allodynia, mast cell and neutrophil infiltration, nitrotyrosine formation, NGF, iNOS, IL1β, MCP1, inhibited NF-kB pathway
**ECSWT**				
**Acute IC models**				
Chen et al., 2014 [18]	SD rats (male), 325-350g, adult, n=18	CYP (150 mg/kg), i.p.	ECSWT (200 pulses, 0.11 mJ/mm^2 skin), surface above the urinary bladder, 3 and 24h after CYP	reduced micturition, urothelial injury, proteinuria, hematuria, infiltration of inflammatory cells, IL6, IL12, TNFα, NF-κB, MMP9, RANTES, iNOS, NOX-1, CD74, CD68, MIF, Cox-2, substance P
**Chronic IC models**				
Li et al., 2019 [66]	BALB/c mice (female), 5 weeks old, n=40	UPK3A (200 μg), s.c., at 6 weeks of age, booster dose at 10 weeks of age	ECSWT (400 pulses, 0,09 mJ/mm^2), skin of the pelvic region, 3x/week	increased pain threshold, improved bladder function, decreased urinary frequency, TNFα, NGF

Wang et al., 2017 [67]	SD rats (female), 250-300g, n= 33	CYP (75 mg/kg), i.p., day 1 and 4	ECSWT (300 pulses, 0.12 mJ/mm^2)	reduced pain behavior, bladder overactivity, inflammation, NGF, IL6 COX2
**TARGETING PAR/PURINERGIC RECEPTORS/TRP CHANNELS**				
**Acute IC models**				
Monjotin et al., 2016 [19]	SD rats Wistar Han rats (female), 200–250g, n=76	CYP (150 mg/kg), i.p.	selective PAR1 agonist [F166357] (30 uM), intravesically, 1h	restored the physiological urodynamic profile and mean voided volume, reduced micturition frequency, increased individual voided volumes
Kouzoukas et al., 2015 [68]	C57BL/6 mice (female), 13 weeks, n=52	PAR-activating peptide [TFLLR-NH2, AYPGKF-NH2] (100 uM, 150 ul), intravesically, 1h	MIF antagonist [ISO-1] (20 mg/kg), intravesically, 15 min pretreatment	prevented PAR4- and reduced PAR1-induced mechanical hypersensitivity, reduced MIF
Kouzoukas et al., 2016 [69]	C57BL/6 mice (female), 13-17 weeks	AYPGKF-NH2 (100 μM, 150 μl), intravesically, 1h	ISO-1 (20 mg/kg), i.p., HMGB1 antagonist [glycyrrhizin] (50 mg/kg), i.p.	prevented mechanical hypersensitivity
Ma et al., 2019 [70]	C57/BL6 (male&female)	CYP (300 mg/kg), i.p.	ISO-1 (20 mg/kg), i.p., 10 min pre-/24h post-treatment	increase micturition volume, reduced micturition frequency and bladder inflammation
Irie et al., 2020 [71]	ddY mice (female), 18-22g, 4–5 weeks, n=38	substance P (6 nmol, 200 μL), intravesically, 24h	anti-HMGB1 Ab (1 mg/kg, i.p., 20 μg, intravesically)	reduced referred hyperalgesia/allodynia, bladder swelling
Tanaka et al., 2014 [72]	ddY mice (female), 18-22g, 4-5 weeks	CYP (150 mg/kg), i.p.	rhsTM (0.1 - 10 mg/kg), i.p., anti-HMGB1 Ab (1 mg/kg) i.p., 30 min pretreatment	prevented CYP-induced nociceptive behaviour
Beckel et al., 2015 [73]	SD rats (female), n=45; Long-Evans rats (female), n=3, 225-275g	LPS (100 ug/ml), intravesically	Pannexin 1 channels antagonist [BB-FCF] (1-100 μM)	decreased ATP
Arronson et al., 2012 [74]	SD rats (male), 300–400g, n=54	CYP (100 mg/kg), i.p.	P1A1 rec. ant. [DPCPX] (1mg/kg)/P1A2B rec. ant. [PSB11 15] (1 mg/kg)/P2 rec. ant. [suramin] (10 mg/kg), i.p., 1x/day, 5 days pretreatment	DPCPX suppressed submucosal thickening, muscarinic M5 receptor, detrusor mast cell infiltration
**Chronic IC models**				
Yang et al., 2021 [20]	SD rats (female), 250g	CYP (75 mg/kg), i.p., 1x/3 days, 7 days.	A2a rec. ant. [ZM241385] (10 μg), intrathecal injection	prevented bladder overactivity and hyperalgesia
Ko et al., 2021 [75]	SD rats (female), 240-260g, 12 weeks, n = 40	CYP (75 mg/kg), i.p., 1x/3 days, 9 days	A2a rec. agonist [PDRN] (8 mg/kg), i.p., 1x/day	decreased contraction pressure and time, inflammatory score, TNFα, IL1β, IκB-α phosphorylation, suppressed NF-κB and MAPK

				pathway, inhibited DNA fragmentation, increased ICI
deBerry et al., 2015 [76]	C57BL/6 mice (female), 8-12 weeks	CYP (100 mg/kg), i.p., 1x/2 days, 5 days	α-artemin (10 mg/kg), i.p., 30 min pre-/post-treatment	blocked and reversed mechanical hyperalgesia, decreased TRPA1 and ERK phosphorylation
Merrill et al., 2014 [77]	Wistar rats (male), 300-350g, adult	repeated variate stress (7 days)	TRPV4 antagonist [HC067047] (1 μM), intravesically, 30 min	Improved bladder capacity and ICI, increased voided volume
**Acute and chronic IC models**				
Kawasaki et al., 2021 [78]	C57BL6 mice (male), 5-6 weeks	acute: CYP (300 mg/kg), i.p. chronic: CYP (150 mg/kg), i.p., 4 days	TRPV4 ant. [compound X] (0.03 - 10 mg/kg), [GSK 2193874] (50 mg/kg), p.o.	reduced nociceptive pain, mechanical hypersensitivity
**TARGETING microRNA**				
**Acute IC models**				
Hou et al., 2020 [79]	SD rats (female), n=70	CYP (100 mg/kg) i.p., PS (10 mg/kg), LPS (2 mg/kg), intravesically	miR-495 mimic (4 mg/kg plasmids), i.p., 10 D	decreased mast cell count, fibrosis, IL6, IL8, IL10, IL-7, TNFα, JAK3
Song et al. 2019 [80]	SD rats (female), 250-300g, 12 weeks, n=70	PS (10 mg/ml) intravesically, 45 min, LPS (750 μg/ml), intravesically., 30 min; repeated after 24h	miR-132 inhibitor (5 μl), i.p./JAK-STAT inhibitor [AG490] (5 μl), i.p.	reduced inflammatory cell infiltration, fibrosis, mast cell numbers, IL6, IL10, IFNγ, TNFα, ICAM1, improved bladder capacity, basal and peak pressure
**TARGETING THE CANNABINOID SYSTEM**				
**Acute IC models**				
Wang et al., 2013 [81]	C57BL/6NH mice (female), 10-12 weeks	acrolein (1 mM), intravesically	CB2 agonist [GP1a] (1-10 mg/kg), s.c., 10 min pretreatment	decreased bladder weight and mechanical sensitivity, inhibited phosphorylation of ERK1/2
Wang et al., 2014 [82]	C57BL/6NH mice (female), 10-12 weeks	acrolein (0.5 mM), intravesically, 40 min	CB2 agonist [GP1a] (10 mg/kg), i.p.	decreased bladder weight, edema, peripheral sensitivity to mechanical stimuli, number of urine spots
Tambaro et al., 2014 [83]	CD-1 mice (male), 25-35g	LPS (25 mg/kg), i.p.	CB2 agonist [JWH015] (5 mg/kg), i.p., 3 min pretreatment/4h post-treatment	reduced MPO activity, leukocyte infiltration, IL1α, IL1β, and TNFα
Berger et al., 2019 [84]	CD-1 mice (female), 27-33g, n=29 for i.p. LPS BALB/c mice (female), 17-23g, n=22 for intravesical LPS	LPS (20 mg/kg), i.p./LPS (150 μg/ml), intravesically	CB2 agonist [HU308] (5 mg/kg), i.p. 30 min post-treatment/ CB2 agonist [BCP] (100 mg/kg), intravesically, 30 min post treatment/BCP (100 mg/kg), p.o., 1h pretreatment	reduced number of adherent leukocytes in bladder venules, inflammation, mechanical allodynia

Pessina et al., 2015 [85]	Wistar rats (female), 200-240g, n=132	CYP (200 mg/kg), i.p.	CB1 and CB2 agonist [palmitoylethanolamide] (5-20 mg/kg), i.p., 30 min pretreatment	attenuated pain behavior and reduced number of voiding episodes
Liu et al., 2020 [86]	C57BL/6J mice (female), 10-13 weeks	CYP (150 mg/kg), i.p.	selective CB2 agonist [JWH-133] (1 mg/kg), i.p., 30 min pretreatment	reduced mechanical hyperalgesia, number of void spots, IL1β, TNFα, IL8, oxidative stress, increased autophagy
**OTHER THERAPEUTIC AGENTS and TARGETS**				
**Acute IC models**				
Shimizu et al., 2013 [87]	SD rats (female), 200-250g, 11 weeks	HCl (0.4 M), intravesically, 90 s	hydroxyfasudil (10 mg/kg), i.p., 1x/day, 7 days	increased ICI, reduced edema, neutrophil infiltration, cellular proliferation, inhibited RhoA/ROCK signaling
de Oliveira et al., 2016 [88]	C57BL/6 mice (female), 20-25g, 10 weeks	CYP (300 mg/kg), i.p.	sGC activator [BAY 58-2667] (1 mg/kg), p.o., pretreatment	increased micturition volume, normalized basal pressure, voiding frequency, NVC, ICI, reduced ROS
Liu et al., 2015 [89]	SD rats (male), 6-8 weeks	CYP (150 mg/kg), i.p.	NMDAR antagonists [MK-801] (3 mg/kg)/[AP5] (5 mg/kg), i.v./PI3K inhibitor [LY294002] (50 μg/kg), i.v.	reduced bladder hypertrophy, voiding frequency, and urine output, increased ICI
Lai et al., 2017 [90]	C57BL/6J mice (female), 18-23g, 8-10 weeks, n=27	CYP (150 mg/kg), i.p.	anti-VEGF antibodies (10 mg/kg), i.p., 36 h pretreatment	reduced pelvic hypersensitivity
Chen et al., 2020 [91]	SD rats (female), 225-250g, 6-8 weeks, n=30	CYP (150 mg/kg), i.p.	platelet-rich plasma (0.3 ml) alone or in combination with HA (1 mg/ml), intravesically, 1 h	increased voiding interval, ZO-1, reduced bladder edema and IL6
Majima et al., 2017 [92]	SD rats (female), 9 weeks	H_2O_2 (1.5%) intravesically, 10 min	liposomes with NGF antisense oligonucleotide [OND] (12 μM), intravesically	reduced ICI, bladder weight, nociceptive behavior, inflammatory cell infiltration, NGF and TRPV1
Hu et al., 2005 [93]	Wistar rats (female), adult	CYP (150 mg/kg), i.p.	recombinant NGF sequestering protein trkA Ig2 [REN1820] (200 μg), i.v. on day 1 and 2	decreased bladder overactivity (ICI, NVC, decreased voiding frequency), absent changes in behavior
Tyagi et al., 2006 [94]	SD rats, 150-200g	CYP (100 mg/kg), i.p.	NGF antisense [TAT-PNA: peptide nucleic acid, conjugated to TAT protein] (100 μM), intravesically, 30 min pretreatment	reduced bladder contraction frequency, NGF

Coelho et al., 2015 [23]	SD rats (female), 250-300g, n=66	CYP (150 mg/kg), i.p.	TrkA antagonist [GW441756] (0.5 mg), i.p., 24 h pretreatment and 1x/day	prevented pain behavior and mechanical hyperalgesia, NGF
**Chronic IC models**				
Zeybek et al., 2007 [95]	Wistar rats (female), 200-250g, adult	water avoidance stress, 2h/day, 5 days	taurine (50 mg/kg), i.p., pretreatment	retained urothelial integrity, decreased mast cell count, MDA, increased GSH
Benigni et al., 2006 [96]	BALB/c mice (female), 18-20g, 8 weeks	immunisation with chicken ovalbumin (10 μg/animal) i.p. 1x/week, 4 weeks, ovalbumin (1 mg/150 μl) intravesically, 2x for 30 min	vitamin D3 analogue [BXL628] (30, 75 μg/kg), p.o., 1x/day, 8-12 days	reduced edema, mast cell, eosinophil and lymphomononuclear cell numbers, IL13, MMCP4, FcεRIα
Akin et al., 2015 [97]	SD rats (female), 8 weeks	PS (5 mg/ml), intravesically, 2x/day, 3 days	hydroxyfasudil (10 mg/kg), i.p.	reduced micturition frequency, inflammation, degeneration, LOX, normalized urine volume, increased GSH, CAT and SOD activity
Zhang et al, 2016 [98]	SD rats (female), 250-300g	CYP (150 mg/kg), i.p., PS (30 mg/ml, 0.5 ml), intravesically, 30 min, LPS (2 mg/ml), intravesically, 45 min; repeated on day 3	anti-ICAM antibody (0,5 mg/ml), i.p., 1 h post-treatment, day 1 and 3	decreased inflammation grade and mast cell count, reduced expression of P2X2/P2X3 receptors, PGE2, EP1/EP2 receptors, NK1R, TNFα and ICAM1
Minami et al., 2019 [99]	ICR mice (female), 6 weeks, n=16	H_2O_2 (1.5 %), intravesically, 20 min, repeated on days 1 and 3	hyperbaric oxygen (hyperbaric chamber, pressure 0.2 ATA, 100% O_2 inflow), 30 min on days 4 and 7	improved urinary frequency and tidal voiding volume, reduced bladder weight, edema, leukocyte infiltration, vasodilatation, urothelial permeability, IL6, IL1β, TNFα, TRPV1, TRPV4, fibrosis, increased eNOS
Mahal et al., 2018 [100]	SD rats (female), 6 weeks, n=24	CYP (50 mg/kg), i.p., 2x/week, 2 weeks	PPAR-γ agonist [pioglitazone] (15 mg/kg), gastric gavage, 1x/day, 2 weeks	improved bladder function, reduced urinary frequency, increased cystometric capacity, improved urothelial structural integrity

Supplementary Table S3: Methodological quality and reported measures undertaken to avoid bias. A 12-point quality control checklist was generated based on published ARRIVE guidelines describing the minimum information that all scientific publications reporting research using animals should include.

Methodological quality

1. Number of experimental and control groups

2. Number of animals

3. Species and strain

4. Sex of the animals

5. Age of the animals

6. Weight of the animals

7. Housing and husbandry

8. Drug formulation, dose, site and route of administration

Risk of bias:

9. Sample size calculation

10. Randomization into groups

11. Blinded caretaker/investigator

12. Blinded assessment of outcome

	**METHODOLOGICAL QUALITY**								**RISK OF BIAS**				
Reference	1	2	3	4	5	6	7	8	9	10	11	12	SCORE
	**Agents recommended by AUA guidelines to treat IC/BPS**												
Hauser et al., 2009 [24]	no	no	yes	yes	no	no	no	yes	no	no	no	no	3
Yeh et al., 2010 [25]	yes	yes	yes	yes	no	yes	no	yes	no	no	no	no	6
Greenwood et al., 2018 [26]	no	no	yes	yes	no	yes	no	yes	no	no	no	no	4
Jensen et al., 2019 [27]	yes	yes	yes	yes	yes	no	no	yes	no	no	no	no	6
Towner et al., 2020 [28]	no	no	yes	yes	no	no	no	yes	no	no	no	no	3
Lee et al., 2013 [6]	yes	yes	yes	yes	yes	no	no	yes	no	no	no	no	6
Engles et al., 2013 [29]	no	no	yes	no	no	no	no	yes	no	no	no	no	2
Ottamasathien et al., 2011 [30]	yes	yes	yes	yes	yes	no	no	yes	no	no	no	no	6

Rajasekaran et al., 2006 [7]	yes	yes	yes	yes	no	yes	no	yes	no	no	no	no	6
Soler et al., 2008 [31]	yes	yes	yes	yes	no	yes	no	yes	no	no	no	no	6
Smith et al., 2005 [32]	yes	yes	yes	yes	no	yes	no	yes	no	no	no	no	6
Chuang et al., 2009 [33]	yes	yes	yes	yes	no	yes	no	yes	no	no	no	no	6
Kim et al., 2011 [34]	no	no	yes	yes	yes	no	no	yes	no	no	no	no	4
	**Agents approved to treat other diseases**												
Boucher et al., 2008 [35]	yes	yes	yes	yes	no	yes	no	yes	no	no	no	no	6
Funahashi et al., 2014 [36]	yes	yes	yes	yes	no	yes	no	yes	no	no	no	yes	7
Zhang et al., 2017 [12]	yes	yes	yes	yes	yes	yes	no	yes	no	no	no	no	7
Boudieu et al., 2019 [13]	yes	yes	yes	yes	no	yes	no	yes	no	no	no	no	6
Ichihara et al., 2017 [37]	no	no	yes	yes	no	yes	no	yes	no	no	no	no	4
Cetinel et al., 2010 [38]	yes	yes	yes	yes	no	yes	yes	yes	no	no	no	yes	8
Holschneider et al., 2020 [39]	no	yes	yes	yes	no	no	yes	yes	no	no	no	no	5
Bicer et al., 2014 [40]	yes	yes	yes	yes	yes	no	no	yes	no	no	no	no	6
Liu et al., 2019 [41]	yes	yes	yes	yes	yes	no	no	yes	no	yes	no	yes	8
Yoshizumi et al., 2021 [42]	no	no	yes	yes	no	yes	no	yes	no	no	no	no	4
	**Other intravesical therapy and improved drug delivery systems**												
Chuang et al., 2003 [43]	yes	yes	yes	yes	no	yes	no	yes	no	no	no	no	6
Gonzales et al., 2005 [44]	no	yes	yes	yes	yes	no	no	yes	no	no	no	no	5
Tyagi et al., 2008 [45]	no	no	yes	yes	no	yes	no	yes	no	no	no	no	4
Fraser et al., 2003 [46]	yes	yes	yes	yes	no	yes	no	yes	no	no	no	no	6
Tyagi et al., 2009 [47]	yes	yes	yes	yes	no	yes	no	yes	no	yes	no	no	7
Konkol et al., 2015 [48]	yes	yes	yes	yes	yes	no	no	yes	no	no	no	yes	7
	**Stem cell therapy**												
Song et al., 2015 [49]	yes	yes	yes	yes	yes	no	no	yes	no	no	no	no	6

Hirose et al., 2016 [50]	yes	yes	yes	yes	no	yes	no	yes	no	no	yes	no	7
Kim et al., 2017 [51]	yes	yes	yes	yes	yes	no	no	yes	no	yes	yes	yes	10
Li et al., 2017 [52]	yes	yes	yes	yes	no	yes	no	yes	no	yes	no	yes	8
Xie et al., 2018 [14]	yes	yes	yes	yes	no	yes	no	yes	no	no	no	no	6
Kim et al., 2020 [53]	yes	yes	yes	yes	yes	no	no	yes	no	no	no	no	6
Inoue et al., 2019 [54]	no	no	yes	yes	yes	no	no	yes	no	no	no	no	4
Ryu et al., 2018 [55]	no	no	yes	yes	yes	no	no	yes	no	no	no	no	4
Furuta et al., 2018 [56]	yes	yes	yes	yes	no	yes	no	yes	no	no	no	no	6
Chung et al., 2020 [57]	yes	yes	yes	yes	yes	no	no	yes	no	no	no	no	7
Lee et al., 2018 [58]	yes	yes	yes	yes	yes	no	no	yes	no	no	no	no	6
	**Plant-based therapy**												
Wang et al., 2020 [15]	yes	yes	yes	yes	no	yes	yes	yes	no	no	no	no	7
Wang et al., 2021 [16]	yes	yes	yes	yes	no	yes	yes	yes	no	no	no	no	7
Nassir et al., 2019 [59]	yes	yes	yes	yes	yes	yes	yes	yes	no	yes	no	no	9
Bazi et al., 2012 [60]	yes	yes	yes	yes	no	yes	no	yes	no	yes	no	yes	8
Li et al., 2020 [61]	yes	yes	yes	yes	no	yes	no	yes	no	yes	no	yes	8
Luo et al., 2020 [62]	yes	yes	yes	yes	no	yes	no	yes	no	yes	no	no	7
Shih et al., 2021 [63]	yes	yes	yes	yes	yes	yes	no	yes	no	no	no	no	7
Liu et al., 2021 [64]	yes	yes	yes	yes	yes	yes	no	yes	no	yes	no	no	8
Ostardo et al., 2018 [65]	yes	yes	yes	yes	no	yes	no	yes	no	yes	no	yes	8
	**ECSWT**												
Chen et al., 2014 [18]	yes	yes	yes	yes	no	yes	no	yes	no	yes	no	no	7
Li et al., 2019 [66]	yes	yes	yes	yes	yes	no	no	yes	no	no	no	no	6
Wang et al., 2017 [67]	yes	yes	yes	yes	no	yes	no	yes	no	no	yes	no	7
	**Targeting PAR/purinergic receptors/TRP channels**												
Monjotin et al., 2016 [19]	yes	yes	yes	yes	no	yes	yes	yes	yes	yes	no	yes	10

Kouzoukas et al., 2015 [68]	yes	yes	yes	yes	yes	no	no	yes	no	no	no	yes	7
Kouzoukas et al., 2016 [69]	yes	no	yes	yes	yes	no	no	yes	no	no	no	yes	6
Ma et al., 2019 [70]	no	no	yes	yes	no	no	no	yes	no	no	yes	no	4
Irie et al., 2020 [71]	yes	yes	yes	yes	yes	yes	no	yes	no	no	no	no	7
Tanaka et al., 2014 [72]	no	no	yes	yes	yes	yes	no	yes	no	no	no	no	5
Beckel et al., 2015 [73]	no	yes	yes	yes	no	yes	no	yes	no	no	no	no	5
Aronsson et al., 2012 [74]	no	yes	yes	yes	no	yes	no	yes	no	no	no	no	5
Yang et al., 2021 [20]	no	no	yes	yes	no	no	no	yes	no	no	no	no	3
Ko et al., 2021 [75]	yes	yes	yes	yes	yes	yes	no	yes	no	yes	no	no	8
DeBerry et al., 2015 [76]	no	no	yes	yes	yes	no	no	yes	no	yes	no	yes	6
Merrill et al., 2014 [77]	yes	yes	yes	yes	no	yes	no	yes	no	no	no	no	6
Kawasaki et al., 2021 [78]	no	no	yes	yes	yes	no	no	yes	no	no	no	no	4
	**Targeting microRNAs**												
Hou et al., 2021 [79]	yes	yes	no	yes	no	no	no	yes	no	no	no	no	4
Song et al., 2019 [80]	yes	yes	yes	yes	yes	yes	no	yes	no	yes	no	no	8
	**Targeting the cannabinoid system**												
Wang et al., 2013 [81]	yes	yes	yes	yes	yes	no	no	yes	no	no	no	yes	7
Wang et al., 2014 [82]	no	yes	yes	yes	yes	no	no	yes	no	no	no	yes	6
Tambaro et al., 2014 [83]	no	yes	yes	yes	no	yes	yes	yes	no	no	no	no	6
Berger et al., 2019 [84]	yes	yes	yes	yes	no	yes	no	yes	no	no	no	no	6
Pessina et al., 2015 [85]	no	yes	yes	yes	no	yes	yes	yes	no	no	no	no	6
Liu et al., 2019 [86]	yes	yes	yes	yes	yes	no	no	yes	no	yes	no	no	7
	**Other therapeutic agents and targets**												
Shimizu et al., 2013 [87]	yes	yes	yes	yes	yes	yes	no	yes	no	yes	no	no	8
de Oliveira et al., 2016 [88]	yes	yes	yes	yes	yes	yes	no	yes	no	no	no	no	7

Liu et al., 2015 [89]	no	yes	yes	yes	yes	no	no	yes	no	no	no	no	5
Lai et al., 2017 [90]	yes	yes	yes	yes	yes	yes	no	yes	no	no	no	no	7
Chen et al., 2020 [91]	yes	yes	yes	yes	yes	yes	no	yes	no	yes	no	no	8
Majima et al., 2017 [92]	yes	yes	yes	yes	yes	no	no	yes	no	no	no	yes	7
Hu et al., 2005 [93]	yes	yes	yes	yes	no	no	no	yes	no	no	no	no	5
Tyagi et al., 2006 [94]	no	no	yes	no	no	yes	no	no	no	no	no	no	2
Coelho et al., 2014 [23]	yes	yes	yes	yes	no	yes	no	yes	no	no	no	yes	7
Zeybek et al., 2007 [95]	no	no	yes	yes	no	yes	no	yes	no	no	no	no	4
Benigni et.al., 2006 [96]	yes	yes	yes	yes	yes	yes	no	yes	no	no	no	yes	8
Akin et al., 2015 [97]	yes	yes	yes	yes	yes	yes	no	yes	no	no	no	no	8
Zhang et al., 2016 [98]	yes	yes	yes	yes	no	yes	no	yes	no	no	no	no	6
Minami et al., 2018 [99]	yes	yes	yes	yes	yes	no	no	yes	no	no	no	no	6
Mahal et al., 2018 [100]	yes	yes	yes	yes	yes	no	no	yes	no	no	no	no	6
Total (n)	63	70	88	86	41	53	8	88	1	18	4	18	

References

1. Rooney, P.; Srivastava, A.; Watson, L.; Quinlan, L.R.; Pandit, A. Hyaluronic acid decreases IL-6 and IL-8 secretion and permeability in an inflammatory model of interstitial cystitis. *Acta Biomater* **2015**, *19*, 66-75, doi:10.1016/j.actbio.2015.02.030.
2. Stellavato, A.; Pirozzi, A.V.A.; Diana, P.; Reale, S.; Vassallo, V.; Fusco, A.; Donnarumma, G.; De Rosa, M.; Schiraldi, C. Hyaluronic acid and chondroitin sulfate, alone or in combination, efficiently counteract induced bladder cell damage and inflammation. *PLoS One* **2019**, *14*, e0218475, doi:10.1371/journal.pone.0218475.
3. Rozenberg, B.B.; Janssen, D.A.W.; Jansen, C.F.J.; Schalken, J.A.; Heesakkers, J. Improving the barrier function of damaged cultured urothelium using chondroitin sulfate. *Neurourol Urodyn* **2020**, *39*, 558-564, doi:10.1002/nau.24240.
4. Rooney, P.; Ryan, C.; McDermott, B.J.; Dev, K.; Pandit, A.; Quinlan, L.R. Effect of Glycosaminoglycan Replacement on Markers of Interstitial Cystitis In Vitro. *Front Pharmacol* **2020**, *11*, 575043, doi:10.3389/fphar.2020.575043.
5. Rooney, P.R.; Kannala, V.K.; Kotla, N.G.; Benito, A.; Dupin, D.; Loinaz, I.; Quinlan, L.R.; Rochev, Y.; Pandit, A. A high molecular weight hyaluronic acid biphasic dispersion as potential therapeutics for interstitial cystitis. *J Biomed Mater Res B Appl Biomater* **2021**, *109*, 864-876, doi:10.1002/jbm.b.34751.
6. Lee, W.Y.; Savage, J.R.; Zhang, J.X.; Jia, W.J.; Oottamasathien, S.; Prestwich, G.D. Prevention of Anti-microbial Peptide LL-37-Induced Apoptosis and ATP Release in the Urinary Bladder by a Modified Glycosaminoglycan. *Plos One* **2013**, *8*, doi:10.1371/journal.pone.0077854.
7. Rajasekaran, M.; Stein, P.; Parsons, C.L. Toxic factors in human urine that injure urothelium. *Int J Urol* **2006**, *13*, 409-414, doi:10.1111/j.1442-2042.2006.01301.x.
8. Melchior, D.; Packer, C.S.; Johnson, T.C.; Kaefer, M. Dimethyl sulfoxide: does it change the functional properties of the bladder wall? *J Urol* **2003**, *170*, 253-258, doi:10.1097/01.ju.0000071520.73686.3d.
9. Rapp, D.E.; Turk, K.W.; Bales, G.T.; Cook, S.P. Botulinum toxin type a inhibits calcitonin gene-related peptide release from isolated rat bladder. *J Urol* **2006**, *175*, 1138-1142, doi:10.1016/s0022-5347(05)00322-8.
10. Lucioni, A.; Bales, G.T.; Lotan, T.L.; McGehee, D.S.; Cook, S.P.; Rapp, D.E. Botulinum toxin type A inhibits sensory neuropeptide release in rat bladder models of acute injury and chronic inflammation. *BJU Int* **2008**, *101*, 366-370, doi:10.1111/j.1464-410X.2007.07312.x.
11. Grundy, L.; Caldwell, A.; Garcia Caraballo, S.; Erickson, A.; Schober, G.; Castro, J.; Harrington, A.M.; Brierley, S.M. Histamine induces peripheral and central hypersensitivity to bladder distension via the histamine H(1) receptor and TRPV1. *Am J Physiol Renal Physiol* **2020**, *318*, F298-f314, doi:10.1152/ajprenal.00435.2019.
12. Zhang, X.L.; Gao, S.; Tanaka, M.; Zhang, Z.; Huang, Y.R.; Mitsui, T.; Kamiyama, M.; Koizumi, S.; Fan, J.L.; Takeda, M., et al. Carbenoxolone inhibits TRPV4 channel-initiated oxidative urothelial injury and ameliorates cyclophosphamide-induced bladder dysfunction. *Journal of Cellular and Molecular Medicine* **2017**, *21*, 1791-1802, doi:10.1111/jcmm.13100.
13. Boudieu, L.; Mountadem, S.; Lashermes, A.; Meleine, M.; Ulmann, L.; Rassendren, F.; Aissouni, Y.; Sion, B.; Carvalho, F.A.; Ardid, D. Blocking α(2)δ-1 Subunit Reduces Bladder Hypersensitivity and Inflammation in a Cystitis Mouse Model by Decreasing NF-kB Pathway Activation. *Front Pharmacol* **2019**, *10*, 133, doi:10.3389/fphar.2019.00133.
14. Xie, J.; Liu, B.; Chen, J.; Xu, Y.; Zhan, H.; Yang, F.; Li, W.; Zhou, X. Umbilical cord-derived mesenchymal stem cells alleviated inflammation and inhibited apoptosis in interstitial cystitis via AKT/mTOR signaling pathway. *Biochem Biophys Res Commun* **2018**, *495*, 546-552, doi:10.1016/j.bbrc.2017.11.072.
15. Wang, X.; Fan, L.; Yin, H.; Zhou, Y.; Tang, X.; Fei, X.; Tang, H.; Peng, J.; Ren, X.; Xue, Y., et al. Protective effect of Aster tataricus extract on NLRP3-mediated pyroptosis of bladder urothelial cells. *J Cell Mol Med* **2020**, *24*, 13336-13345, doi:10.1111/jcmm.15952.
16. Wang, X.; Yin, H.; Fan, L.; Zhou, Y.Q.; Tang, X.L.; Fei, X.J.; Tang, H.L.; Peng, J.; Zhang, J.J.; Xue, Y., et al. Shionone alleviates NLRP3 inflammasome mediated pyroptosis in interstitial cystitis injury. *International Immunopharmacology* **2021**, *90*, doi:10.1016/j.intimp.2020.107132.
17. Liu, M.; Xu, Y.F.; Feng, Y.; Yang, F.Q.; Luo, J.; Zhai, W.; Che, J.P.; Wang, G.C.; Zheng, J.H. Epigallocatechin gallate attenuates interstitial cystitis in human bladder urothelium cells by modulating purinergic receptors. *J Surg Res* **2013**, *183*, 397-404, doi:10.1016/j.jss.2012.11.041.

18. Chen, Y.T.; Yang, C.C.; Sun, C.K.; Chiang, H.J.; Chen, Y.L.; Sung, P.H.; Zhen, Y.Y.; Huang, T.H.; Chang, C.L.; Chen, H.H., et al. Extracorporeal shock wave therapy ameliorates cyclophosphamide-induced rat acute interstitial cystitis though inhibiting infammation and oxidative stress-in vitro and in vivo experiment studies. *American Journal of Translational Research* **2014**, *6*, 631-648.
19. Monjotin, N.; Gillespie, J.; Farrié, M.; Le Grand, B.; Junquero, D.; Vergnolle, N. F16357, a novel protease-activated receptor 1 antagonist, improves urodynamic parameters in a rat model of interstitial cystitis. *British Journal of Pharmacology* **2016**, 10.1111/bph.13501, 2224-2236, doi:10.1111/bph.13501.
20. Yang, Y.; Zhang, H.; Lu, Q.; Liu, X.; Fan, Y.; Zhu, J.; Sun, B.; Zhao, J.; Dong, X.; Li, L. Suppression of adenosine A(2a) receptors alleviates bladder overactivity and hyperalgesia in cyclophosphamide-induced cystitis by inhibiting TRPV1. *Biochem Pharmacol* **2021**, *183*, 114340, doi:10.1016/j.bcp.2020.114340.
21. Hayn, M.H.; Ballesteros, I.; de Miguel, F.; Coyle, C.H.; Tyagi, S.; Yoshimura, N.; Chancellor, M.B.; Tyagi, P. Functional and immunohistochemical characterization of CB1 and CB2 receptors in rat bladder. *Urology* **2008**, *72*, 1174-1178, doi:10.1016/j.urology.2008.03.044.
22. Keay, S.; Kaczmarek, P.; Zhang, C.O.; Koch, K.; Szekely, Z.; Barchi, J.J., Jr.; Michejda, C. Normalization of proliferation and tight junction formation in bladder epithelial cells from patients with interstitial cystitis/painful bladder syndrome by d-proline and d-pipecolic acid derivatives of antiproliferative factor. *Chem Biol Drug Des* **2011**, *77*, 421-430, doi:10.1111/j.1747-0285.2011.01108.x.
23. Coelho, A.; Wolf-Johnston, A.S.; Shinde, S.; Cruz, C.D.; Cruz, F.; Avelino, A.; Birder, L.A. Urinary bladder inflammation induces changes in urothelial nerve growth factor and TRPV1 channels. *Br J Pharmacol* **2015**, *172*, 1691-1699, doi:10.1111/bph.12958.
24. Hauser, P.J.; Buethe, D.A.; Califano, J.; Sofinowski, T.M.; Culkin, D.J.; Hurst, R.E. Restoring barrier function to acid damaged bladder by intravesical chondroitin sulfate. *J Urol* **2009**, *182*, 2477-2482, doi:10.1016/j.juro.2009.07.013.
25. Yeh, C.H.; Chiang, H.S.; Chien, C.T. Hyaluronic acid ameliorates bladder hyperactivity via the inhibition of H2O2-enhanced purinergic and muscarinic signaling in the rat. *Neurourol Urodyn* **2010**, *29*, 765-770, doi:10.1002/nau.20830.
26. Greenwood-Van Meerveld, B.; Mohammadi, E.; Latorre, R.; Truitt, E.R., 3rd; Jay, G.D.; Sullivan, B.D.; Schmidt, T.A.; Smith, N.; Saunders, D.; Ziegler, J., et al. Preclinical Animal Studies of Intravesical Recombinant Human Proteoglycan 4 as a Novel Potential Therapy for Diseases Resulting From Increased Bladder Permeability. *Urology* **2018**, *116*, 230.e231-230.e237, doi:10.1016/j.urology.2018.02.034.
27. Jensen, M.M.; Jia, W.; Schults, A.J.; Isaacson, K.J.; Steinhauff, D.; Green, B.; Zachary, B.; Cappello, J.; Ghandehari, H.; Oottamasathien, S. Temperature-responsive silk-elastinlike protein polymer enhancement of intravesical drug delivery of a therapeutic glycosaminoglycan for treatment of interstitial cystitis/painful bladder syndrome. *Biomaterials* **2019**, *217*, 119293, doi:10.1016/j.biomaterials.2019.119293.
28. Towner, R.A.; Greenwood-Van Meerveld, B.; Mohammadi, E.; Saunders, D.; Smith, N.; Sant, G.R.; Shain, H.C.; Jozefiak, T.H.; Hurst, R.E. SuperGAG biopolymers for treatment of excessive bladder permeability. *Pharmacol Res Perspect* **2021**, *9*, e00709, doi:10.1002/prp2.709.
29. Engles, C.D.; Hauser, P.J.; Abdullah, S.N.; Culkin, D.J.; Hurst, R.E. Intravesical chondroitin sulfate inhibits recruitment of inflammatory cells in an acute acid damage "leaky bladder" model of cystitis. *Urology* **2012**, *79*, 483.e413-487, doi:10.1016/j.urology.2011.10.010.
30. Oottamasathien, S.; Jia, W.J.; McCoard, L.; Slack, S.; Zhang, J.X.; Skardal, A.; Job, K.; Kennedy, T.P.; Dull, R.O.; Prestwich, G.D. A Murine Model of Inflammatory Bladder Disease: Cathelicidin Peptide Induced Bladder Inflammation and Treatment With Sulfated Polysaccharides. *Journal of Urology* **2011**, *186*, 1684-1692, doi:10.1016/j.juro.2011.03.099.
31. Soler, R.; Bruschini, H.; Truzzi, J.C.; Martins, J.R.; Camara, N.O.; Alves, M.T.; Leite, K.R.; Nader, H.B.; Srougi, M.; Ortiz, V. Urinary glycosaminoglycans excretion and the effect of dimethyl sulfoxide in an experimental model of non-bacterial cystitis. *Int Braz J Urol* **2008**, *34*, 503-511; discussion 511, doi:10.1590/s1677-55382008000400013.
32. Smith, C.P.; Vemulakonda, V.M.; Kiss, S.; Boone, T.B.; Somogyi, G.T. Enhanced ATP release from rat bladder urothelium during chronic bladder inflammation: Effect of botulinum toxin A. *Neurochemistry International* **2005**, *47*, 291-297, doi:10.1016/j.neuint.2005.04.021.

33. Chuang, Y.C.; Yoshimura, N.; Huang, C.C.; Wu, M.; Chiang, P.H.; Chancellor, M.B. Intravesical botulinum toxin A administration inhibits COX-2 and EP4 expression and suppresses bladder hyperactivity in cyclophosphamide-induced cystitis in rats. *Eur Urol* **2009**, *56*, 159-166, doi:10.1016/j.eururo.2008.05.007.
34. Kim, R.; Liu, W.; Chen, X.; Kreder, K.J.; Luo, Y. Intravesical dimethyl sulfoxide inhibits acute and chronic bladder inflammation in transgenic experimental autoimmune cystitis models. *J Biomed Biotechnol* **2011**, *2011*, 937061, doi:10.1155/2011/937061.
35. Boucher, W.; Stern, J.M.; Kotsinyan, V.; Kempuraj, D.; Papaliodis, D.; Cohen, M.S.; Theoharides, T.C. Intravesical nanocrystalline silver decreases experimental bladder inflammation. *J Urol* **2008**, *179*, 1598-1602, doi:10.1016/j.juro.2007.11.037.
36. Funahashi, Y.; Yoshida, M.; Yamamoto, T.; Majima, T.; Takai, S.; Gotoh, M. Intravesical application of rebamipide promotes urothelial healing in a rat cystitis model. *J Urol* **2014**, *192*, 1864-1870, doi:10.1016/j.juro.2014.06.081.
37. Ichihara, K.; Aizawa, N.; Akiyama, Y.; Kamei, J.; Masumori, N.; Andersson, K.E.; Homma, Y.; Igawa, Y. Toll-like receptor 7 is overexpressed in the bladder of Hunner-type interstitial cystitis, and its activation in the mouse bladder can induce cystitis and bladder pain. *Pain* **2017**, *158*, 1538-1545, doi:10.1097/j.pain.0000000000000947.
38. Çetinel, S.; Çanıllıoğlu, Y.E.; Çikler, E.; Sener, G.; Ercan, F. Leukotriene D4 receptor antagonist montelukast alleviates protamine sulphate-induced changes in rat urinary bladder. *BJU Int* **2011**, *107*, 1320-1325, doi:10.1111/j.1464-410X.2010.09532.x.
39. Holschneider, D.P.; Wang, Z.; Chang, H.Y.; Zhang, R.; Gao, Y.L.; Guo, Y.M.; Mao, J.; Rodriguez, L.V. Ceftriaxone inhibits stress-induced bladder hyperalgesia and alters cerebral micturition and nociceptive circuits in the rat: A multidisciplinary approach to the study of urologic chronic pelvic pain syndrome research network study. *Neurourology and Urodynamics* **2020**, *39*, 1628-1643, doi:10.1002/nau.24424.
40. Bicer, F.; Altuntas, C.Z.; Izgi, K.; Ozer, A.; Kavran, M.; Tuohy, V.K.; Daneshgari, F. Chronic pelvic allodynia is mediated by CCL2 through mast cells in an experimental autoimmune cystitis model. *Am J Physiol Renal Physiol* **2015**, *308*, F103-113, doi:10.1152/ajprenal.00202.2014.
41. Liu, B.K.; Jin, X.W.; Lu, H.Z.; Zhang, X.; Zhao, Z.H.; Shao, Y. The Effects of Neurokinin-1 Receptor Antagonist in an Experimental Autoimmune Cystitis Model Resembling Bladder Pain Syndrome/Interstitial Cystitis. *Inflammation* **2019**, *42*, 246-254, doi:10.1007/s10753-018-0888-2.
42. Yoshizumi, M.; Watanabe, C.; Mizoguchi, H. Gabapentin reduces painful bladder hypersensitivity in rats with lipopolysaccharide-induced chronic cystitis. *Pharmacol Res Perspect* **2021**, *9*, e00697, doi:10.1002/prp2.697.
43. Chuang, Y.C.; Chou, A.K.; Wu, P.C.; Chiang, P.H.; Yu, T.J.; Yang, L.C.; Yoshimura, N.; Chancellor, M.B. Gene therapy for bladder pain with gene gun particle encoding pro-opiomelanocortin cDNA. *J Urol* **2003**, *170*, 2044-2048, doi:10.1097/01.ju.0000092945.76827.47.
44. Gonzalez, R.R.; Fong, T.; Belmar, N.; Saban, M.; Felsen, D.; Te, A. Modulating bladder neuro-inflammation: RDP58, a novel anti-inflammatory peptide, decreases inflammation and nerve growth factor production in experimental cystitis. *J Urol* **2005**, *173*, 630-634, doi:10.1097/01.ju.0000143192.68223.f7.
45. Tyagi, P.; Chancellor, M.; Yoshimura, N.; Huang, L. Activity of different phospholipids in attenuating hyperactivity in bladder irritation. *BJU Int* **2008**, *101*, 627-632, doi:10.1111/j.1464-410X.2007.07334.x.
46. Fraser, M.O.; Chuang, Y.C.; Tyagi, P.; Yokoyama, T.; Yoshimura, N.; Huang, L.; De Groat, W.C.; Chancellor, M.B. Intravesical liposome administration--a novel treatment for hyperactive bladder in the rat. *Urology* **2003**, *61*, 656-663, doi:10.1016/s0090-4295(02)02281-1.
47. Tyagi, P.; Hsieh, V.C.; Yoshimura, N.; Kaufman, J.; Chancellor, M.B. Instillation of liposomes vs dimethyl sulphoxide or pentosan polysulphate for reducing bladder hyperactivity. *BJU Int* **2009**, *104*, 1689-1692, doi:10.1111/j.1464-410X.2009.08673.x.
48. Konkol, Y.; Bernoulli, J.; Streng, T.; Jääskeläinen, K.; Laihia, J.; Leino, L. Intravesical treatment with cis-urocanic acid improves bladder function in rat model of acute bladder inflammation. *Neurourol Urodyn* **2016**, *35*, 786-791, doi:10.1002/nau.22818.
49. Song, M.; Lim, J.; Yu, H.Y.; Park, J.; Chun, J.Y.; Jeong, J.; Heo, J.; Kang, H.; Kim, Y.; Cho, Y.M., et al. Mesenchymal Stem Cell Therapy Alleviates Interstitial Cystitis by Activating Wnt Signaling Pathway. *Stem Cells and Development* **2015**, *24*, 1648-1657, doi:10.1089/scd.2014.0459.
50. Hirose, Y.; Yamamoto, T.; Nakashima, M.; Funahashi, Y.; Matsukawa, Y.; Yamaguchi, M.; Kawabata, S.; Gotoh, M. Injection of Dental Pulp Stem Cells Promotes Healing of Damaged

Bladder Tissue in a Rat Model of Chemically Induced Cystitis. *Cell Transplant* **2016**, *25*, 425-436, doi:10.3727/096368915x689523.
51. Kim, A.; Yu, H.Y.; Lim, J.; Ryu, C.M.; Kim, Y.H.; Heo, J.; Han, J.Y.; Lee, S.; Bae, Y.S.; Kim, J.Y., et al. Improved efficacy and in vivo cellular properties of human embryonic stem cell derivative in a preclinical model of bladder pain syndrome. *Sci Rep* **2017**, *7*, 8872, doi:10.1038/s41598-017-09330-x.
52. Li, J.; Luo, H.; Dong, X.; Liu, Q.; Wu, C.; Zhang, T.; Hu, X.; Zhang, Y.; Song, B.; Li, L. Therapeutic effect of urine-derived stem cells for protamine/lipopolysaccharide-induced interstitial cystitis in a rat model. *Stem Cell Res Ther* **2017**, *8*, 107, doi:10.1186/s13287-017-0547-9.
53. Kim, B.S.; Chun, S.Y.; Lee, E.H.; Chung, J.W.; Lee, J.N.; Ha, Y.S.; Choi, J.Y.; Song, P.H.; Kwon, T.G.; Han, M.H., et al. Efficacy of combination therapy with pentosan polysulfate sodium and adipose tissue-derived stem cells for the management of interstitial cystitis in a rat model. *Stem Cell Res* **2020**, *45*, 101801, doi:10.1016/j.scr.2020.101801.
54. Inoue, Y.; Kishida, T.; Kotani, S.I.; Akiyoshi, M.; Taga, H.; Seki, M.; Ukimura, O.; Mazda, O. Direct conversion of fibroblasts into urothelial cells that may be recruited to regenerating mucosa of injured urinary bladder. *Sci Rep* **2019**, *9*, 13850, doi:10.1038/s41598-019-50388-6.
55. Ryu, C.M.; Yu, H.Y.; Lee, H.Y.; Shin, J.H.; Lee, S.; Ju, H.; Paulson, B.; Lee, S.; Kim, S.; Lim, J., et al. Longitudinal intravital imaging of transplanted mesenchymal stem cells elucidates their functional integration and therapeutic potency in an animal model of interstitial cystitis/bladder pain syndrome. *Theranostics* **2018**, *8*, 5610-5624, doi:10.7150/thno.27559.
56. Furuta, A.; Yamamoto, T.; Igarashi, T.; Suzuki, Y.; Egawa, S.; Yoshimura, N. Bladder wall injection of mesenchymal stem cells ameliorates bladder inflammation, overactivity, and nociception in a chemically induced interstitial cystitis-like rat model. *Int Urogynecol J* **2018**, *29*, 1615-1622, doi:10.1007/s00192-018-3592-8.
57. Chung, J.W.; Chun, S.Y.; Lee, E.H.; Ha, Y.S.; Lee, J.N.; Song, P.H.; Yoo, E.S.; Kwon, T.G.; Chung, S.K.; Kim, B.S. Verification of mesenchymal stem cell injection therapy for interstitial cystitis in a rat model. *PLoS One* **2019**, *14*, e0226390, doi:10.1371/journal.pone.0226390.
58. Lee, S.W.; Ryu, C.M.; Shin, J.H.; Choi, D.; Kim, A.; Yu, H.Y.; Han, J.Y.; Lee, H.Y.; Lim, J.; Kim, Y.H., et al. The Therapeutic Effect of Human Embryonic Stem Cell-Derived Multipotent Mesenchymal Stem Cells on Chemical-Induced Cystitis in Rats. *Int Neurourol J* **2018**, *22*, S34-45, doi:10.5213/inj.1836014.007.
59. Nassir, A.M.; Ibrahim, I.A.A.; Afify, M.A.; ElSawy, N.A.; Imam, M.T.; Shaheen, M.H.; Basyuni, M.A.; Bader, A.; Azhar, R.A.; Shahzad, N. Olea europaea subsp. Cuspidata and Juniperus procera Hydroalcoholic Leaves' Extracts Modulate Stress Hormones in Stress-Induced Cystitis in Rats. *Urological Science* **2019**, *30*, 151-156, doi:10.4103/uros.Uros_130_18.
60. Bazi, T.; Hajj-Hussein, I.A.; Awwad, J.; Shams, A.; Hijaz, M.; Jurjus, A. A modulating effect of epigallocatechin gallate (EGCG), a tea catechin, on the bladder of rats exposed to water avoidance stress. *Neurourol Urodyn* **2013**, *32*, 287-292, doi:10.1002/nau.22288.
61. Li, W.; Yang, F.; Zhan, H.; Liu, B.; Cai, J.; Luo, Y.; Zhou, X. Houttuynia cordata Extract Ameliorates Bladder Damage and Improves Bladder Symptoms via Anti-Inflammatory Effect in Rats with Interstitial Cystitis. *Evid Based Complement Alternat Med* **2020**, *2020*, 9026901, doi:10.1155/2020/9026901.
62. Luo, J.; Yang, C.; Luo, X.; Yang, Y.; Li, J.; Song, B.; Zhao, J.; Li, L. Chlorogenic acid attenuates cyclophosphamide-induced rat interstitial cystitis. *Life Sci* **2020**, *254*, 117590, doi:10.1016/j.lfs.2020.117590.
63. Shih, H.J.; Chang, C.Y.; Lai, C.H.; Huang, C.J. Therapeutic effect of modulating the NLRP3-regulated transforming growth factor-β signaling pathway on interstitial cystitis/bladder pain syndrome. *Biomedicine and Pharmacotherapy* **2021**, *138*, doi:10.1016/j.biopha.2021.111522.
64. Liu, Y.C.; Lee, W.T.; Liang, C.C.; Lo, T.S.; Hsieh, W.C.; Lin, Y.H. Beneficial effect of Bletilla striata extract solution on zymosan-induced interstitial cystitis in rat. *Neurourology and Urodynamics* **2021**, *40*, 763-770, doi:10.1002/nau.24630.
65. Ostardo, E.; Impellizzeri, D.; Cervigni, M.; Porru, D.; Sommariva, M.; Cordaro, M.; Siracusa, R.; Fusco, R.; Gugliandolo, E.; Crupi, R., et al. Adelmidrol plus sodium hyaluronate in IC/BPS or conditions associated to chronic urothelial inflammation. A translational study. *Pharmacological Research* **2018**, *134*, 16-30, doi:10.1016/j.phrs.2018.05.013.
66. Li, H.; Zhang, Z.; Peng, J.; Xin, Z.; Li, M.; Yang, B.; Fang, D.; Tang, Y.; Guo, Y. Treatment with low-energy shock wave alleviates pain in an animal model of uroplakin 3A-induced

autoimmune interstitial cystitis/painful bladder syndrome. *Investig Clin Urol* **2019**, *60*, 359-366, doi:10.4111/icu.2019.60.5.359.
67. Wang, H.J.; Lee, W.C.; Tyagi, P.; Huang, C.C.; Chuang, Y.C. Effects of low energy shock wave therapy on inflammatory moleculars, bladder pain, and bladder function in a rat cystitis model. *Neurourol Urodyn* **2017**, *36*, 1440-1447, doi:10.1002/nau.23141.
68. Kouzoukas, D.E.; Meyer-Siegler, K.L.; Ma, F.; Westlund, K.N.; Hunt, D.E.; Vera, P.L. Macrophage Migration Inhibitory Factor Mediates PAR-Induced Bladder Pain. *PLoS One* **2015**, *10*, e0127628, doi:10.1371/journal.pone.0127628.
69. Kouzoukas, D.E.; Ma, F.; Meyer-Siegler, K.L.; Westlund, K.N.; Hunt, D.E.; Vera, P.L. Protease-Activated Receptor 4 Induces Bladder Pain through High Mobility Group Box-1. *PLoS One* **2016**, *11*, e0152055, doi:10.1371/journal.pone.0152055.
70. Bosco, G.; Ostardo, E.; Rizzato, A.; Garetto, G.; Paganini, M.; Melloni, G.; Giron, G.; Pietrosanti, L.; Martinelli, I.; Camporesi, E. Clinical and morphological effects of hyperbaric oxygen therapy in patients with interstitial cystitis associated with fibromyalgia. *BMC Urol* **2019**, *19*, 108, doi:10.1186/s12894-019-0545-6.
71. Irie, Y.; Tsubota, M.; Maeda, M.; Hiramoto, S.; Sekiguchi, F.; Ishikura, H.; Wake, H.; Nishibori, M.; Kawabata, A. HMGB1 and its membrane receptors as therapeutic targets in an intravesical substance P-induced bladder pain syndrome mouse model. *J Pharmacol Sci* **2020**, *143*, 112-116, doi:10.1016/j.jphs.2020.03.002.
72. Tanaka, J.; Yamaguchi, K.; Ishikura, H.; Tsubota, M.; Sekiguchi, F.; Seki, Y.; Tsujiuchi, T.; Murai, A.; Umemura, T.; Kawabata, A. Bladder pain relief by HMGB1 neutralization and soluble thrombomodulin in mice with cyclophosphamide-induced cystitis. *Neuropharmacology* **2014**, *79*, 112-118, doi:10.1016/j.neuropharm.2013.11.003.
73. Beckel, J.M.; Daugherty, S.L.; Tyagi, P.; Wolf-Johnston, A.S.; Birder, L.A.; Mitchell, C.H.; de Groat, W.C. Pannexin 1 channels mediate the release of ATP into the lumen of the rat urinary bladder. *Journal of Physiology-London* **2015**, *593*, 1857-1871, doi:10.1113/jphysiol.2014.283119.
74. Aronsson, P.; Johnsson, M.; Vesela, R.; Winder, M.; Tobin, G. Adenosine receptor antagonism suppresses functional and histological inflammatory changes in the rat urinary bladder. *Auton Neurosci* **2012**, *171*, 49-57, doi:10.1016/j.autneu.2012.10.006.
75. Ko, I.G.; Jin, J.J.; Hwang, L.; Kim, S.H.; Kim, C.J.; Won, K.Y.; Na, Y.G.; Kim, K.H.; Kim, S.J. Adenosine A(2A) Receptor Agonist Polydeoxyribonucleotide Alleviates Interstitial Cystitis-Induced Voiding Dysfunction by Suppressing Inflammation and Apoptosis in Rats. *J Inflamm Res* **2021**, *14*, 367-378, doi:10.2147/jir.S287346.
76. DeBerry, J.J.; Saloman, J.L.; Dragoo, B.K.; Albers, K.M.; Davis, B.M. Artemin Immunotherapy Is Effective in Preventing and Reversing Cystitis-Induced Bladder Hyperalgesia via TRPA1 Regulation. *J Pain* **2015**, *16*, 628-636, doi:10.1016/j.jpain.2015.03.014.
77. Merrill, L.; Vizzard, M.A. Intravesical TRPV4 blockade reduces repeated variate stress-induced bladder dysfunction by increasing bladder capacity and decreasing voiding frequency in male rats. *Am J Physiol Regul Integr Comp Physiol* **2014**, *307*, R471-480, doi:10.1152/ajpregu.00008.2014.
78. Kawasaki, S.; Soga, M.; Sakurai, Y.; Nanchi, I.; Yamamoto, M.; Imai, S.; Takahashi, T.; Tsuno, N.; Asaki, T.; Morioka, Y., et al. Selective blockade of transient receptor potential vanilloid 4 reduces cyclophosphamide-induced bladder pain in mice. *Eur J Pharmacol* **2021**, 10.1016/j.ejphar.2021.174040, 174040, doi:10.1016/j.ejphar.2021.174040.
79. Hou, Y.; Li, H.; Huo, W. MicroRNA-495 alleviates ulcerative interstitial cystitis via inactivating the JAK-STAT signaling pathway by inhibiting JAK3. *Int Urogynecol J* **2021**, 10.1007/s00192-020-04593-x, doi:10.1007/s00192-020-04593-x.
80. Song, Y.J.; Cao, J.Y.; Jin, Z.; Hu, W.G.; Wu, R.H.; Tian, L.H.; Yang, B.; Wang, J.; Xiao, Y.; Huang, C.B. Inhibition of microRNA-132 attenuates inflammatory response and detrusor fibrosis in rats with interstitial cystitis via the JAK-STAT signaling pathway. *J Cell Biochem* **2019**, *120*, 9147-9158, doi:10.1002/jcb.28190.
81. Wang, Z.Y.; Wang, P.; Bjorling, D.E. Activation of cannabinoid receptor 2 inhibits experimental cystitis. *Am J Physiol Regul Integr Comp Physiol* **2013**, *304*, R846-853, doi:10.1152/ajpregu.00585.2012.
82. Wang, Z.Y.; Wang, P.; Bjorling, D.E. Treatment with a cannabinoid receptor 2 agonist decreases severity of established cystitis. *J Urol* **2014**, *191*, 1153-1158, doi:10.1016/j.juro.2013.10.102.
83. Tambaro, S.; Casu, M.A.; Mastinu, A.; Lazzari, P. Evaluation of selective cannabinoid CB(1) and CB(2) receptor agonists in a mouse model of lipopolysaccharide-induced interstitial cystitis. *Eur J Pharmacol* **2014**, *729*, 67-74, doi:10.1016/j.ejphar.2014.02.013.

84. Berger, G.; Arora, N.; Burkovskiy, I.; Xia, Y.F.; Chinnadurai, A.; Westhofen, R.; Hagn, G.; Cox, A.; Kelly, M.; Zhou, J., et al. Experimental Cannabinoid 2 Receptor Activation by Phyto-Derived and Synthetic Cannabinoid Ligands in LPS-Induced Interstitial Cystitis in Mice. *Molecules* **2019**, *24*, doi:10.3390/molecules24234239.
85. Pessina, F.; Capasso, R.; Borrelli, F.; Aveta, T.; Buono, L.; Valacchi, G.; Fiorenzani, P.; Di Marzo, V.; Orlando, P.; Izzo, A.A. Protective effect of palmitoylethanolamide in a rat model of cystitis. *J Urol* **2015**, *193*, 1401-1408, doi:10.1016/j.juro.2014.11.083.
86. Liu, Q.; Wu, Z.; Liu, Y.; Chen, L.; Zhao, H.; Guo, H.; Zhu, K.; Wang, W.; Chen, S.; Zhou, N., et al. Cannabinoid receptor 2 activation decreases severity of cyclophosphamide-induced cystitis via regulating autophagy. *Neurourol Urodyn* **2020**, *39*, 158-169, doi:10.1002/nau.24205.
87. Shimizu, N.; De Velasco, M.A.; Umekawa, T.; Uemura, H.; Yoshikawa, K. Effects of the Rho kinase inhibitor, hydroxyfasudil, on bladder dysfunction and inflammation in rats with HCl-induced cystitis. *Int J Urol* **2013**, *20*, 1136-1143, doi:10.1111/iju.12119.
88. de Oliveira, M.G.; Calmasini, F.B.; Alexandre, E.C.; De Nucci, G.; Mónica, F.Z.; Antunes, E. Activation of soluble guanylyl cyclase by BAY 58-2667 improves bladder function in cyclophosphamide-induced cystitis in mice. *Am J Physiol Renal Physiol* **2016**, *311*, F85-93, doi:10.1152/ajprenal.00041.2016.
89. Liu, M.; Shen, S.; Kendig, D.M.; Mahavadi, S.; Murthy, K.S.; Grider, J.R.; Qiao, L.Y. Inhibition of NMDAR reduces bladder hypertrophy and improves bladder function in cyclophosphamide induced cystitis. *J Urol* **2015**, *193*, 1676-1683, doi:10.1016/j.juro.2014.12.092.
90. Lai, H.H.; Shen, B.; Vijairania, P.; Zhang, X.; Vogt, S.K.; Gereau, R.W.t. Anti-vascular endothelial growth factor treatment decreases bladder pain in cyclophosphamide cystitis: a Multidisciplinary Approach to the Study of Chronic Pelvic Pain (MAPP) Research Network animal model study. *BJU Int* **2017**, *120*, 576-583, doi:10.1111/bju.13924.
91. Chen, Y.H.; Man, K.M.; Chen, W.C.; Liu, P.L.; Tsai, K.S.; Tsai, M.Y.; Wu, Y.T.; Chen, H.Y. Platelet-Rich Plasma Ameliorates Cyclophosphamide-Induced Acute Interstitial Cystitis/Painful Bladder Syndrome in a Rat Model. *Diagnostics (Basel)* **2020**, *10*, doi:10.3390/diagnostics10060381.
92. Majima, T.; Tyagi, P.; Dogishi, K.; Kashyap, M.; Funahashi, Y.; Gotoh, M.; Chancellor, M.B.; Yoshimura, N. Effect of Intravesical Liposome-Based Nerve Growth Factor Antisense Therapy on Bladder Overactivity and Nociception in a Rat Model of Cystitis Induced by Hydrogen Peroxide. *Hum Gene Ther* **2017**, *28*, 598-609, doi:10.1089/hum.2016.121.
93. Hu, V.Y.; Zvara, P.; Dattilio, A.; Redman, T.L.; Allen, S.J.; Dawbarn, D.; Stroemer, R.P.; Vizzard, M.A. Decrease in bladder overactivity with REN1820 in rats with cyclophosphamide induced cystitis. *J Urol* **2005**, *173*, 1016-1021, doi:10.1097/01.ju.0000155170.15023.e5.
94. Tyagi, P.; Banerjee, R.; Basu, S.; Yoshimura, N.; Chancellor, M.; Huang, L. Intravesical antisense therapy for cystitis using TAT-peptide nucleic acid conjugates. *Mol Pharm* **2006**, *3*, 398-406, doi:10.1021/mp050093x.
95. Zeybek, A.; Sağlam, B.; Cikler, E.; Cetinel, S.; Ercan, F.; Sener, G. Taurine ameliorates stress-induced degeneration of the urinary bladder. *Acta Histochem* **2007**, *109*, 208-214, doi:10.1016/j.acthis.2006.12.001.
96. Benigni, F.; Baroni, E.; Zecevic, M.; Zvara, P.; Streng, T.; Hedlund, P.; Colli, E.; D'Ambrosio, D.; Andersson, K.E. Oral treatment with a vitamin D3 analogue (BXL628) has anti-inflammatory effects in rodent model of interstitial cystitis. *BJU Int* **2006**, *97*, 617-624, doi:10.1111/j.1464-410X.2006.05971.x.
97. Akin, Y.; Bozkurt, A.; Erol, H.S.; Halici, M.; Celebi, F.; Kapakin, K.A.; Gulmez, H.; Ates, M.; Coban, A.; Nuhoglu, B. Impact of Rho-Kinase Inhibitor Hydroxyfasudil in Protamine Sulphate Induced Cystitis Rat Bladder. *Low Urin Tract Symptoms* **2015**, *7*, 108-114, doi:10.1111/luts.12058.
98. Zhang, X.; He, H.; Lu, G.; Xu, T.; Qin, L.; Wang, X.; Jin, X.; Liu, B.; Zhao, Z.; Shen, Z., et al. Specific inhibition of ICAM-1 effectively reduces bladder inflammation in a rat model of severe non-bacterial cystitis. *Sci Rep* **2016**, *6*, 35672, doi:10.1038/srep35672.
99. Minami, A.; Tanaka, T.; Otoshi, T.; Kuratsukuri, K.; Nakatani, T. Hyperbaric oxygen significantly improves frequent urination, hyperalgesia, and tissue damage in a mouse long-lasting cystitis model induced by an intravesical instillation of hydrogen peroxide. *Neurourol Urodyn* **2019**, *38*, 97-106, doi:10.1002/nau.23822.
100. Mahal, A.; Young-Lin, N.; Dobberfuhl, A.; Estes, J.; Comiter, C.V. Peroxisome proliferator-activated receptor gamma agonist as a novel treatment for interstitial cystitis: A rat model. *Investig Clin Urol* **2018**, *59*, 257-262, doi:10.4111/icu.2018.59.4.257.
